# Characteristic flavor metabolic network of fish sauce microbiota with different fermentation processes based on metagenomics

**DOI:** 10.3389/fnut.2023.1121310

**Published:** 2023-03-06

**Authors:** Jiarun Han, Tao Kong, Jialan Jiang, Xin Zhao, Xilian Zhao, Ping Li, Qing Gu

**Affiliations:** Key Laboratory for Food Microbial Technology of Zhejiang Province, College of Food Science and Biotechnology, Zhejiang Gongshang University, Hangzhou, Zhejiang, China

**Keywords:** fish sauce, fermentation processes, flavor information, key enzymes, key microorganisms, metabolic network

## Abstract

This article purposed to discuss the connection between microbiota and characteristic flavor of different fish sauces (Natural fermentation (WQ), koji outdoor fermentation (YQ), heat preservation with enzyme (BWE), and heat preservation with koji (BWQ)) at the early (3 months) and late stage (7 months). A total of 117 flavor compounds were determined according to SPME-GC–MS analysis. O2PLS-DA and VIP values were used to reveal 15 and 28 flavor markers of different fish sauces at 3 and 7 M of fermentation. Further, the possible flavor formation pathways were analyzed using metagenomic sequencing, and the key microbes associated with flavor formation were identified at the genetic level. The top 10 genera related to flavor generation, such as *Lactobacillus*, *Staphylococcus*, *Enterobacter*, etc., appeared to play a prominent part in the flavor formation of fish sauce. The difference was that only BWQ and BWE groups could produce ethyl-alcohol through amino acid metabolism, while YQ, BWE and BWQ groups could generate phenylacetaldehyde through the transformation of Phe by α-ketoacid decarboxylase and aromatic amino acid transferase. Our research contributes to clarifying the various metabolic roles of microorganisms in the flavor generation of fish sauce.

## 1. Introduction

Fish sauce is a unique flavored condiment with amber-color, which is traditionally fermented from low-value marine fish in coastal areas. It is consumed and produced in numerous regions of the world, such as Southeast Asia, the southeast coast of China, South Korea, Japan and Europe (1). Fish sauce, as the traditional seasoning proposed by “A bite of China,” contains a variety of proteins, amino acids, minerals and vitamins with high nutritional value (2). Natural fermentation is regarded as the extension and inheritance of traditional fish sauce processing. However, due to the open fermentation environment and long production cycle (6–18 months) of traditional fermented fish sauce, food manufacturers are looking for alternative fish sauce fermentation technologies (3). Therefore, the fast-fermented technology has gradually become the research hotspot in the fish sauce production.

At present, the commercial rapid production of fish sauce mainly includes koji fermentation, microbial fermentation, compound protease fermentation and heat preservation fermentation. It has been reported that adding starter cultures could better regulate the fermentation process of fermented fish and hopefully achieve the quality standardization (4). For example, mixed kojis (the ratio of *Aspergillus oryzae* to *Aspergillus niger* was 3:1) were utilized to ferment fish sauce prepared by freshwater fish by-products, which could expedite the fermentation process and increase the generation of taste-and flavor-inducing compounds (5). Adding enzyme (cathepsin, chymotrypsin or trypsin) or enzyme-rich fish viscera to raw ingredients could speed up the breakdown of fish protein and shorten the fish sauce fermentation period (6). Moreover, there are also several fermentation progresses based on the combination of different approaches. Therefore, the selection of reliable rapid-fermentation method is of great significance for the fish sauce production.

In the fermentation process of fish sauce, microorganisms play a vital role. The enzymes associate with the formation of flavor are mainly derived from microorganisms, and the production of characteristic flavor substances is primarily affected by the complex microbial metabolic activities (7). With the fermentation goes on, carbohydrate hydrolysis, protein hydrolysis and lipid hydrolysis produced vital flavor or flavor precursors under the action of multiple enzymes that were secreted in microbial metabolic pathways, and diversified beneficial microorganisms and key enzymes in those pathways exert a vital role in the sensory characteristics and flavor formation of final fish sauce (8). In order to targeted promote the accumulation of characteristic flavor substances in fish sauce, it is necessary to clarify the potential generation pathways of characteristic flavor substances and the specific role of microbiota in flavor formation pathways, and select appropriate flavor-related microorganisms as starter culture. In recent years, the relationship between microbial succession and volatile compounds of naturally fermented fish sauce has been studied according to bidirectional orthogonal partial least squares (O2PLS) model (9). However, the inferences about how microorganisms influence the flavor generation of different rapid-fermented fish sauces required to be further expanded.

Metagenomic is an efficient technique to thoroughly unveil the metabolic capacity of microorganisms, which has been widely used in studying microbial metabolic pathways and screening microbial-specific functional genes (10). This technique was first applied in environment, human (such as skin, digestive tract) and other microbial communities (11), but it has been gradually applied to food microorganisms in recent years. Recently, metagenomic-based techniques have been utilized to study various fermented foods, such as Doubanjiang (12), fermented Suanyu (4) and dark tea (13). For example, Jung et al. clarified that *Leuconostoc mesenteroids* strain mainly participated in carbohydrate metabolism during Korean kimchi fermentation, which further producing lactic acid, ethyl acetate, ethanol and other flavor substances (14). Moreover, ethanol, acetic acid and lactic acid generated during the Shaoxing wine fermentation were also mainly formed through carbohydrate metabolism, while the production of higher alcohols was associated with both amino acid metabolism and carbohydrate metabolism pathways (15). Currently, although some studies have been conducted on fish sauce, there are no reports on the contribution of microbial metabolism to the flavor generation of fish sauce rapid fermentation process.

This study aimed to identify the characteristic flavors of fish sauce and establish the metabolic network of characteristic flavor in different fermentation approaches of fish sauce (Natural fermentation WQ, koji outdoor fermentation YQ, heat preservation with compound enzyme BWE, and heat preservation with koji BWQ). Then, metagenomic techniques were used to annotate the genes encoding the catalytic enzymes involved in the metabolism pathways of flavor substances to the key microorganisms. Eventually, the microorganisms participated in various flavor metabolism pathways were also screened and predicted, which will provide a novel insight into the role of key microorganisms in the characteristic flavor formation of fish sauces.

## 2. Materials and methods

### 2.1. Preparation and sampling of fish sauce with different processes

The fish sauces investigated in our study were collected from Zhejiang Pingtairong Biotechnology Co., Ltd. in Zhoushan, China. The raw anchovies with a body length of 75–140 mm and weight of 5–20 g were selected in the East China Sea. Before fermentation, the whole fish was thawed, washed, minced and placed orderly in the fermentation tank. The following four fermentation processes are performed:

Natural fermentation without koji (WQ): 30% of the sea salt was mixed with the anchovies, and naturally fermented for 7 months at an outdoor temperature environment (20 ± 5) °C.

Koji outdoor fermentation (YQ): Fish sauce mush was first produced by mixing minced fish, deionized water and sea salt at a ratio of 10:5:2 (*w/w/w*). After stirring evenly, the salted fish was further mixed with 20% (*w/w*, koji/fish) soya meal koji, and incubated at 40°C for 30 min, in which the soya meal koji was made from *Aspergillus oryzae* as described by Sun et al. (16). The mixture was then placed in the fermentation tank and continuously fermented for 7 months at the outdoor temperature of (25 ± 5) °C.

Heat preservation fermentation with koji (BWQ): The treatment method of fish samples was similar to that of YQ. After adding koji, the fish sauce was first fermented at 35°C for 5 months. Subsequently, the sample was transferred to a constant temperature incubator at (55 ± 5) °C and continued to be fermented for another 2 months under heat preservation.

Heat preservation fermentation with enzyme (BWE): Minced fish was first added with deionized water at a ratio of 1:1 (g: mL). After adding 0.5% (*w/w*, enzyme/fish) of composite protease and watering bath at 50°C for 2.5 h, 0.7% (*w/w*, enzyme/fish) of flavor protease was mixed with the mixture and incubated at 55°C for 2.5 h. Then, 15% (*w/w*, salt/mixture) of the salt was added and incubated for 5 months in a constant temperature incubator at 35°C. Finally, it was transferred to 45°C for another 2 months under heat preservation fermentation.

The fermentation broth was collected 3 and 7 months after fermentation in a sterilized sampling bottle, and then delivered to the laboratory within 24 h. The fermentation broth was collected for total DNA extraction and metagenomic analysis after filtering with gauze.

### 2.2. Determination of flavor compounds by headspace-solid phase microextraction-gas chromatography–mass spectrometry (HS-SPME/GC/MS)

Fish sauce samples (5 g) was mixed with 100 μL of 2,4,6-trimethylpyridine (0.05 mg/ml) as the internal standard substance in a 20 mL brown sealed vial. After magnetic stirring at 60°C water-bath for 20 min, the SPME fiber assembly (50 μm, DVB/CAR/PDMS, Supelco, Bellafonte, PA, United States) was heated to 250°C for 20 min to eliminate all impurities and inserted into the vial. After extraction at 37°C for 30 min, the SPME fiber was promptly inserted into the GC–MS (7890A-5975C, Agilent, CA, United States) coupled with HP-5MS column (60 m × 250 μm × 0.25 μm). The heating process and MS conditions were described by Zang et al. (8). The volatile compounds were identified according to the NIST 98 with Wiley 275.L library and semi-quantified using internal standard. The relative content of volatile compounds was evaluated by calculating the peak area ratio of each compound with that of the internal standard.

### 2.3. Genomic DNA extraction, library construction and sequencing

The microbial metagenomic DNA extraction was done using a PowerSoil DNA Isolation Kit (MO BIO Laboratories, Carlsbad, CA, United States) on the basis of the protocol described by Hang et al. (17). Briefly, 5 g of different fish sauces were added with 10 mL of aseptic phosphate buffer (PB, 10 mM, pH 7.4) at 4°C for 2 h. The mixtures were then centrifuged at 4°C, 10,000 r/min for 20 min, and the resulting precipitates were utilized for total DNA extraction. After extraction, the purity of genomic DNA was determined using 1% agarose gel electrophoresis. Subsequently, the qualified DNA was entrusted to Biomarker Bioinformatics Technology Co., Ltd. (Beijing, China) for Illumina sequencing.

The qualified genomic DNA was cut into short DNA fragments with a length about 400 bp by using a Covaris M220 nucleic acid shear. Construction of a paired-end (PE) library using a NEXTFLEX^™^ Rapid DNA-Seq Kit (Bioo Scientific Corp.), a bridge PCR was executed through a NovaSeq Reagent Kits/HiSeq X Reagent Kits (Illumina Corp.), and the Illumina platform was used to construct and sequence the qualified libraries. Using Trimmomatic (Illumina Inc.), a quality filtering software, to evaluate the original reads (double-ended sequence) quality, and to obtain the high-quality clean reads. MEGAHIT software (Version 1.1.2) was utilized to assemble the clean reads, and the contig sequences less than 300 bp were screened. QUAST software (Illumina Inc.) was used to evaluate the assembly results. To enhance the reliability and quality of the bioinformatics analysis, Meta Gene Mark software (Version 3.26) was applied in predicting the open reading frames (ORF) of contigs ≥500 bp, while CD-HIT software was utilized to further remove redundant ORF. The SOAPaligner software (Version 1.1.2) was utilized to align the high-quality reads of each sample with the set of non-redundant genes, and the gene abundance information in the corresponding samples was counted. The similarity threshold and coverage threshold were set to 95 and 90%, respectively, and the unigene sequences was obtained using MMseqs2 software.

### 2.4. Bioinformatics analysis and species annotation

The gene set was aligned with the National Center for Biotechnology Information (NCBI) database using the BLASTP command. The species annotation was acquired from the taxonomic information database corresponding to the Non-redundant (NR) library, and the species abundance was then calculated through the sum of the corresponding gene abundance of the species. After obtaining the non-redundant gene set, the unigene sequences were annotated using the functions of the NR database and Kyoto Encyclopedia of Genes and Genomes (KEGG) database based on the unigenes ID, so as to associate the genome sequences with metabolic network and to obtain specific microorganisms involved in the flavor formation pathway of fish sauce.

### 2.5. Multivariate statistical analysis

All samples were performed in triplicate (6 parallel trials of the metagenomic). Statistical analysis and graphing of data were performed using Origin 2018 software. Statistical significance and one-way variance analysis were done by SPSS 26.0 statistical program (IBM Co., Somers, NY, United States). O2PLS-discriminant analysis (O2PLS-DA) and the variable importance in projection (VIP) values were conducted by using SIMCA 14.1 (Umetrics, Sweden).

## 3. Results and discussion

### 3.1. Identification of the main volatile flavor compounds

Flavor is a crucial quality characteristic of fermented food. The primary volatile substances were extracted from different fish sauce samples, and further analyzed by HS-SPME-GC–MS at 3 and 7 months of fermentation. As shown in Table 1, a total of 114 flavor compounds were quantified from eight groups of fish sauces, including 21 aldehydes, 6 alcohols, 19 esters, 6 acids, 35 hydrocarbons, 10 ketones, 14 nitrogen-containing compounds, and 3 sulfur-containing compounds, but the volatile flavor substances were different due to diverse fermentation time-point and fermentation methods. For the four fish sauce samples, the amount and total concentration of volatile flavor compounds were observed to vary and presented an increasing trend over the fermentation period (Supplementary Figure S1). Among them, 14 compounds appeared consistently during the four samples and two fermentation stages. The distinctions in the content and type of volatile compounds are probably on account of the catabolic reactions of flavor precursors during fermentation.

**Table 1:** Volatile flavor compounds detected in different fish sauces using SPME-GC–MS.

RT	Name	Relative volatile content (μg/mL)							
		WQ3M	WQ7M	YQ3M	YQ7M	BWE3M	BWE7M	BWQ3M	BWQ7M
	*Aldehydes*								
1.347	Propanal	0	0	46.92724	74.53048	20.93444	26.16931	0	80.98745
2.514	2-Methylbutanal	25.18379	158.9155	0	0	14.8445	127.2027	23.5959	66.81294
3.996	2-Methyl-2-butenal	0	234.5417	0	0	12.82962	58.48093	34.84767	129.3394
4.345	(E)-2-Pentenal	0	184.9519	0	0	15.9024	65.39804	31.83941	53.82494
5.1072	Isovaleraldehyde	0	34.08896	0	90.24557	0	98.16335	0	135.3719
5.93	Hexanal	279.316	21.9733	201.682	77.745	89.7692	115.0497	214.160	172.0162
7.291	2-Methyl-2-pentenal	0	319.4724	431.0485	0	403.734	0	95.60729	0
8.304	(E)-2-Hexenal	0	180.2459	16.90147	341.3652	60.61842	240.0705	33.59663	180.5515
10.366	Methional	0	0	0	178.8983	0	362.7577	0	385.9792
10.765	Heptanal	143.5336	39.96774	208.3384	79.0592	61.56288	138.0904	147.334	40.27646
14.49	Benzaldehyde	2782.6	27.33964	3130.792	872.8527	2488.5	588.4465	2754.929	116.1127
17.025	Octanal	331.9111	0	381.5959	0	119.2085	0	51.55573	0
17.454	2,4-Heptadienal	0	2090.701	553.1101	2020.521	389.6096	1240.48	65.82912	1258.536
18.793	Phenylacetaldehyde	37.46212	582.4354	629.0618	1092.17	149.4581	628.8384	248.5148	561.917
19.496	(E)-2-Octenal	0	271.5945	152.497	314.2567	61.57298	273.9227	25.69246	314.6937
21.37	Nonanal	18.30522	476.5682	280.1945	518.7482	94.81816	184.4822	47.98468	529.4488
23.09	2,6-Nonadienal	0	369.7108	163.1814	462.8818	66.67757	332.8452	25.08351	336.7473
23.336	4-Ethylbenzaldehyde	25.27285	996.5438	196.4966	1394.483	54.61549	828.2524	0	990.5964
24.784	Decanal	99.65228	145.6045	59.78005	158.2781	20.78829	95.85764	12.21032	125.3513
33.086	Octadecanal	0	39.07641	0	0	0	0	0	0
34.734	Tetradecanal	0	38.69365	0	0	0	0	0	0
	*Alcohols*								
3.8792	Ethanol	3.378143	0	0	18.15189	0	0	0	0
4.986	1-Penten-3-ol	27.46222	169.5122	553.881	984.7008	60.65538	160.2629	34.84886	188.0196
9.151	2-Furanmethanol	0	0	0	86.31507	0	8.386324	0	0
15.9	1-Octen-3-ol	0	357.2402	280.7618	384.9716	77.13581	505.1474	82.17911	282.1227
18.638	Benzyl alcohol	0	148.8238	0	0	0	0	0	0
23.748	trans-1,3-Cyclohexanediol	0	99.10818	0	0	0	0	0	155.7057
	Esters								
4.6901	Ethyl acetate	0	16.77933	0	90.24557	0	0	0	0
8.43	3-Methylbutanoic acid ethyl ester	0	0	0	68.42775	0	0	0	0
10.8065	Methyl caproate	0	101.721	0	404.6704	0	0	0	0
15.583	2-Ethyl-1-hexanol, heptafluorobutyrate	0	428.0361	0	0	117.4069	617.2523	134.3777	0
24.572	Ethyl caprylate	44.23564	263.6055	0	0	0	0	0	0
30.208	Ethyl decanoate	54.60095	291.6033	83.11734	416.6222	0	0	0	0
34.0973	Ethyl laurate	0	96.18709	0	571.2913	0	0	0	0
34.511	Dodecanoic acid ethyl ester	0	131.0084	42.32107	148.5073	0	0	0	0
35.243	Ethyl tridecanoate	0	37.81359	52.92089	14.41412	0	0	0	0
36.439	Tetradecanoic acid ethyl ester	0	474.7651	215.6282	637.0642	0	10.18229	8.968253	71.3057
37.114	Pentadecanoic acid ethyl ester	238.5279	63.54797	51.42748	101.6559	0	0	0	0
37.704	Hexadecanoic acid ethyl ester	0	204.107	0	0	0	11.92417	0	107.4628
38.5862	Methyl myristate	72.27205	0	0	380.818	0	519.0039	0	965.9541
38.676	Ethyl oleate	0	38.19079	15.57832	68.46127	0	0	0	0
40.2843	Methyl benzoate	0	75.05539	0	370.564	0	536.1598	0	351.9567
40.7132	Ethyl myristate	0	13.43951	0	77.01607	0	0	0	0
44.2914	Methyl palmitate	0	120.8609	0	505.7473	0	610.5728	0	445.2535
45.3374	Dibutyl phthalate	0	0	0	696.0264	0	0	0	649.6143
45.9131	Ethyl palmitate	0	68.1373	0	252.0078	0	0	0	0
	*Acids*								
9.884	3-Methylbutanoic acid	0	0	15.03849	58.70234	0	0	0	0
36.256	Tetradecanoic acid	0	300.9691	87.50579	549.267	47.09958	175.138	68.20461	272.6581
37.561	n-Hexadecanoic acid	0	175.4968	6.404423	160.5876	70.92488	288.9303	45.64898	385.7316
38.121	Palmitoleic acid	105.05	257.8971	0	0	0	30.04787	9.609041	85.22667
38.711	Oleic Acid	4.713807	27.59611	9.848375	13.64948	2.821414	0	3.210481	102.064
38.66	Octadecanoic acid	0	0	0	0	0	24.01051	0	4.332157
	Hydrocarbons								
6.496	2,5-Octadiene	90.16907	139.885	76.29479	177.7363	1.88E-07	143.1355	12.48853	78.83587
7.9451	Hexamethylcyclotrisiloxane	0	115.0245	0	539.6035	0	298.0189	0	865.7477
9.575	1,3-cis,5-cis-Octatriene	0	102.4392	0	0	19.5278	26.9498	15.44318	47.44962
10.015	Styrene	0	0	0	0	0	0	27.40336	0
10.656	Nonane	76.4608	158.8402	0	0	0	0	0	0
13.2625	Decane	11.75151	0	0	0	0	189.3898	0	976.6473
13.4213	(Z)-2-Hexene	0	0	0	0	0	0	0	140.6971
18.026	Limonene	0.418361	63.68979	62.65301	388.5096	103.6	231.4555	111.0009	174.4703
18.163	3-Ethyl-2-methyl-1,3-hexadiene	0	0	0	0	0	90.16146	7.086473	115.815
19.0088	Decamethylcyclopentasiloxane	0	10.22425	0	70.85543	0	29.43088	0	34.31943
19.2086	2,3,4-Trimethylhexane	0	210.4314	0	0	0	0	0	0
20.24	1,3-Cyclooctadiene	40.1549	491.6035	261.423	760.7727	127.6798	533.971	73.41383	601.526
20.6716	2-Methylpropyl-cyclohexane	0	99.30607	0	0	0	0	0	0
21.516	3-Ethenyl-cyclopentene	0	833.6355	0	0	0	0	0	0
23.548	1-Methyl-3-(1-methylethenyl)-cyclohexane	0	58.79154	0	0	58.57057	0	0	0
25.264	3-Ethenyl-cyclohexene	0	0	0	0	0	0	27.49809	238.5965
25.3897	Dodecamethylcyclohexasiloxane	0	5.105277	0	33.30191	0	13.3911	0	15.54578
26.718	1,4-Octadiene	0	414.4402	163.4168	497.1908	57.69176	318.043	0	378.9947
27.1994	2,6,11-Trimethyldodecane	0	181.6754	0	0	0	0	0	0
27.341	1-Tridecene	91.37005	182.2684	0	0	22.07765	71.10429	9.941424	87.234
29.664	2,6,10,14-Tetramethylhexadecane	0	171.399	71.30364	103.7624	2.70991	0	0	0
30.294	Tetradecane	18.39554	205.503	82.77528	286.8558	24.74504	141.6939	0	148.575
31.0891	Tetradecamethylcycloheptasiloxane	0	0	0	150.935	0	54.79891	0	42.52823
31.867	Dodecane	10.00184	86.2421	0	132.8437	0	248.0122	0	377.0248
32.32	Cyclopentadecane	0	0	0	89.14576	0	41.96426	0	0
34.568	Hexadecane	53.84665	117.4381	43.42331	151.7209	15.4849	105.7328	20.54664	359.3289
35.444	8-Heptadecene	0	114.7649	21.64626	283.0184	9.41525	440.033	7.962023	955.4925
35.69	Heptadecane	291.1742	1723.803	355.5264	2156.59	147.9926	1109.363	137.8633	1488.065
35.735	2,6,10,14-Tetramethylpentadecane	10.35825	810.3336	286.7821	2793.847	114.7329	684.0448	95.22056	735.4528
36.468	Octadecane	56.10581	149.5219	0	0	0	64.09674	0	0
36.5709	Hexadecamethylcyclooctasiloxane	0	81.04989	0	557.1877	0	193.536	0	134.1758
37	Z-5-Nonadecene	0	68.43229	54.22926	97.34774	0	959.0231	0	67.21958
37.143	Eicosane	27.96351	263.4433	0	43.46325	4.388093	64.65633	4.025814	11.089
41.6475	Octadecamethylcyclononasiloxane	0	0	0	0	0	400.1931	0	122.3712
52.3997	Tetracosamethylcyclododecasiloxane	21.74209	0	0	0	0	59.65333	0	47.12211
	*Ketones*								
4.5785	3-Ethyl-2-pentanone	0	0	0	0	0	118.3791	0	0
12.675	2-Methyl-3-octanone	35.44153	0	0	0	0	0	0	0
14.204	3-Ethylcyclopentanone	213.0454	17.34602	0	0	0	0	0	0
16.172	2,5-Octanedione	0	0	0	0	0	30.77599	0	22.5743
16.7879	2-Nonanone	0	15.91662	0	195.8052	0	582.3603	0	0
19.079	1-(1-Cyclohexen-1-yl)-ethanone	45.51216	106.1929	0	0	7.672029	59.59315	13.33353	77.61854
19.702	Acetophenone	0	150.2189	0	0	6.451766	35.45475	0	0
20.996	3,5-Octadien-2-one	5.057701	1226.141	106.913	0	149.011	1056.084	15.44144	1419.616
24.3673	2-Undecanone	0	34.98604	106.9384	407.4172	31.542	190.8011	16.43305	249.3064
32.731	2-Tridecanone	0	41.56888	21.41429	54.26144	0	3.914102	0	0
	*Nitrogen-containing compounds*								
2.159	Trimethylamine	0	102.573	0	81.02118	8.251684	20.57407	9.892058	19.81494
3.5973	Pentafluoropropionamide	0	0	0	0	0	155.2347	0	0
3.8617	Tetramethylammonium chloride	0	0	0	0	0	159.0332	0	0
24.915	1,3-Benzodioxol-5-amine	0	0	0	25.57346	5.227475	0	4.371592	0
27.542	Indole	49.48112	2340.332	117.9204	126.4935	0	0	1.378218	8.106078
27.5638	3,5-Dimethylpyridine	0	0	0	1640.248	0	0	0	0
3.063	2-Ethylfuran	14.13746	500.7419	0	466.6787	169.9624	736.0765	126.2004	370.6733
8.934	p-Xylene	0	162.3184	0	0	4.600071	31.12435	20.08833	59.1283
11.852	4-Ethylphenol	0	55.66111	33.14721	56.0641	0	39.89269	4.599597	43.44242
12.825	Methoxy-phenyl oxime	0	476.6414	303.5116	818.9987	49.91691	558.6347	83.04874	374.5325
16.378	2-Pentylfuran	0	423.5449	303.0625	501.5835	57.74698	375.9436	0	520.0597
16.916	cis-2-(2-Pentenyl) furan	7.447524	964.6886	640.7324	1115.361	211.9828	763.5381	0	859.0385
24.36	2-n-Heptylfuran	65.98535	171.9864	66.49038	201.3911	9.949272	532.4864	44.7272	303.3025
30.826	Decahydro-4,4,8,9,10-pentamethylnaphthalene	0	36.67291	15.28536	0	0	3.943396	0	18.99096
	*Sulfur-containing compounds*								
1.507	Dimethyl sulfide	0	0	0	0	0	8.872929	0	15.52959
5.4716	(1,1-Dimethylethyl)(1-methylpropyl) disulfide	0	0	0	0	0	209.1847	0	0
12.5458	Dimethyl trisulfide	0	0	0	0	0	359.6799	0	0

Reliability of the identification proposal was indicated by comparing the mass spectra and Kovats index according to literature or compared with NIST98 and Wiley databases. KI Kovats indices were calculated by using an n-alkanes series (C5–C26) under the same chromatographic conditions as the samples. The content (μg/mL) of flavor substance from fish sauces were determined using 2,4,6-trimethylpyridine as internal standard substance. ‘0’ means no detectable.

Aldehydes were the most abundant group with relatively low threshold values, which could contribute to the entirety aroma of fish sauce. Studies have shown that volatile aldehydes are lipid oxidative degradation products, which could conducive to the desirable aroma (malt, grass, cheese, and fruit) as well as the rancid flavor of products (18). Among those aldehydes, the relative concentrations of phenylacetaldehyde, 2,4-heptadienal and octanal reached 629.06, 553.11, and 381.60 μg/mL, respectively, at the early fermentation period of YQ samples (Table 1). Obviously, octadecanal and tetradecanal were only found in WQ groups, while propanal and methional only existed in three other groups of rapid-fermented fish sauces. However, the total content of aldehydes in WQ, BWE and BWQ groups was lower than that in YQ group at different fermentation stages. Notably, benzaldehyde was the predominant volatile component in the early stage of fermentation, and the concentration was significantly higher than in the late stage of the four fish sauce samples. It has been reported that benzaldehyde was mainly generated by the Strecker reaction and possessed a delightful almond flavor (19). Among the aliphatic aldehydes detected in our study, heptanal was the major component that produced the fishy smell. As the fermentation progressed, the content of heptanal significantly decreased except for the BWE samples, indicating that the prolongation of fermentation reduced the astringency of fish sauce. Fatty aldehydes with carbon atoms (>6) were normally ascribed to the oxidation of free fatty acids (FFAs) (20). For example, (E,E)-2,4-decadienal and hexanal had the aroma of meat, while nonanal and octanal originated from oleic acid oxidation contributed to a pleasant aroma (21). The content of hexanal and octanal also decreased in WQ, YQ and BWQ groups during fermentation, and octanal was not even detected in the late fermentation stage, which was probably caused by the changing enzymatic activity of aldehyde-generating microorganisms such as *Enterococcus* and *Lactobacillus*, or the reducing/oxidizing reaction of aldehydes as intermediates into acids or alcohols (22).

A total of 6 kinds of alcohols were detected in different fish sauces, but their contribution to the aroma of fish sauce was not significant because of their high threshold values. It could be seen from Table 1 that the total concentrations of alcohol compounds in four fish sauces increased gradually as fermentation progressed. The alcohols in fish sauce primarily came from the oxidative degradation of polyunsaturated fatty acids, which can also be formed through sugar fermentation and the secondary decomposition of fatty acids hydroperoxide (23). An increase in the contents of ethanol (wine), 1-penten-3-ol (fried onion), and 1-octene-3-ol (mushroom and muddy) during fermentation were observed in four fish sauce groups, but benzyl alcohol (rosy and malty) and trans-1,3-Cyclohexanediol were only found in WQ and BWQ groups, and 2-Furanmethanol (burnt sugar) was only detected in YQ and BWE groups. 1-octene-3-ol was a common flavor substance in aquatic products, which was one of the degradation products of linoleic acid peroxide, and it could be generated by the EMP pathway of carbohydrates and the Ehrlich pathway of amino acids in yeast fermentation (24). Our results were in accordance with Zhao et al. who found that the content of alcohols in *Aspergillus oryzae* koji fermented fish sauce was higher than that in naturally fermented fish sauce (5).

Esters are primarily generated by the esterification of alcohols and short-chain acids. A total of 19 kinds of esters were detected in the four kinds of fish sauces, among which ethyl acetate, methyl caproate, decanoic acid ethyl ester, ethyl laurate, dodecanoic acid ethyl ester, ethyl oleate, ethyl myristate and ethyl palmitate significantly increased and only existed in WQ and YQ groups, which were similar to the main esters in fermented fish “Yucha” (25). Except for ethyl palmitate, other compounds exhibited strong odor intensity, including sweet, floral and winey odors. It was indicated that ethyl acetate and methyl caproate were one of the major flavor compounds in fermented food, which could give a positive wine and fruity aroma to foods (26). Compared with WQ and YQ groups, the content and number of esters in BWQ and BWE groups were relatively low, except for methyl myristate, methyl benzoate and methyl palmitate, which might play a part in the overall flavor of heat preservation fermented fish sauce. Moreover, under the action of numerous microorganisms and complex enzyme systems in *Aspergillus oryzae* koji, the nutrients in raw fish were decomposed and converted into a wide variety of metabolites, and the volatile esters were often more diverse. Liu et al. used GC–MS to compare the volatile components of traditional fish sauce and koji fermented fish sauce, and found that the content of ester compounds in the koji fermentation fish sauce was significantly higher than the commercial fish sauce (27). Therefore, the ester flavor of fish sauce produced by koji fermentation was generally superior to heat preservation and enzyme adding process.

Six acid compounds were detected in four fish sauce samples, and the total content of acids gradually increased at 7 months of fermentation. Volatile acid compounds are produced by hydrolysis or oxidation of fat, most of which have high threshold values and unpleasant odors, such as spoilage taste, thus contributing to an adverse impact on the overall flavor of fish sauce (23). The volatile acids detected in fish sauces were primarily long-chain acids such as tetradecanoic acid, n-hexadecanoic acid, palmitoleic acid, oleic acid and octadecanoic acid, which were also found in marinated carp and traditional Chinese Yulu (28). In addition, several short-chain acids, such as 3-methyl-butanoic acid, only existed in YQ groups, which was manifested as a strong sour and cheesy aroma. It has been reported that volatile acids are mainly formed by amino acids through bacterial fermentation, and lipid oxidation is also related to the formation of some acids (29). In our study, the types and concentrations of acid compounds in BWE and BWQ groups were more abundant than those in WQ group, indicating that the addition of composite protease or koji at the early stage of fermentation could not only rapidly decompose proteins, but also produce large amounts of low molecular volatile acids, which promoted the generation of flavor substances in fish sauce to a certain extent.

Various volatile hydrocarbons have been identified in the volatiles of crustacean fish, which were caused by the homolytic cleavage of fatty acid oxidation free radicals. Most of them are sweet and aromatic, but they have a relatively high threshold and contribute little impact on the overall flavor of fish sauce. There was little difference in the total number of hydrocarbons among four samples, but the total concentration in YQ group was higher than that in other groups, especially at 7 months of fermentation. Among the alkanes identified in our study, eight are long-chain n-alkanes such as nonane, decane, tetradecane, dodecane, hexadecane, heptadecane, octadecane and eicosane. The largest number of long-chain alkanes predominantly came from the cleavage of long-chain fatty acid alkoxy radicals (30). Moreover, four branched-chain alkanes such as 2,6,10,14-tetramethylpentadecane, and 10 cycloalkanes such as cyclopentadecane, were also found in fish sauce samples, which might impart fragrance and sweet flavor to the fish sauces. Some alkenes could form aldehydes and ketones under certain conditions, such as 1-tridecene, 2,5-octadiene and 1,3-cyclooctadiene, which might have a certain impact on the fish sauce flavor. The production of ketones was usually associated with the thermal oxidation of polyunsaturated fatty acids or degradation of amino acids, and ketones appeared to be responsible for fruity and cheesy notes in fish sauce. In ketones, the concentration of 2,5-octanedione and 2-undecanone in YQ, BWQ and BWE groups were significantly higher than that in WQ group. 2,5-Octanedione might have a favorable influence on the overall flavor of fish sauce, and 2-undecanone was reported to be associated with tallow, musty and green notes in fish sauce (24).

Nitrogen-containing compounds in fish sauce were mainly derived from the thermal decomposition of amino acids or the Maillard reaction. Trimethylamine was commonly reported in aquatic products, which usually contributed fishy, baked, roasted and nutty notes to the overall flavor of fish sauce. Trimethylamine could be formed by the degradation of trimethylamine oxide, and was related to bacterial decay, and it could be used as an indicator of aquatic product quality (31). Therefore, heat preservation fermentation could significantly reduce the production of trimethylamine, which was crucial to maintain the quality of fish sauce. Indole was usually associated with flavor quality such as putrefaction and mildew, which was also a common flavor substance in fermented products (32). The formation of indole was closely related to microbial conditions in the fermentation process, and it was an important indicator of product quality. In our study, the contents of indole in BWQ and BWE groups were remarkably lower than that in WQ and YQ groups, which suggested that heat preservation fermentation was an effective way to improve the quality of fish sauce. Further, 2-ethylfuran, p-xylene, 4-ethylphenol, 2-pentulfuran and 2-n-heptylfuran were also detected at different stages of fermentation and showed relatively high concentrations, contributing distinctive odor to different fish sauces. Additionally, sulfur compounds were not detected in almost all samples, except BWE and BWQ groups at the late stage of fermentation. Generally, the formation pathway of volatile sulfur compounds is as follows: methionine generates dimethyl sulfide and methanethiol, which can be further oxidized to dimethyl disulfide and dimethyl trisulfide compounds (33). Our results indicated that the increase of sulfur-containing compounds in fish sauce was slow by adding koji or heat preservation fermentation with koji, thus forming better flavor quality.

Therefore, it can be seen that long-term fermentation of fish sauces can result in an increase in various volatile compounds that affect the flavor. Meanwhile, the flavor of fish sauce not only depends on the fermentation process, but is also closely related to the types of raw materials, microbial species, and fermentation conditions.

### 3.2. Screening of differential flavor compounds in different fermentation process

**Figure 1:**
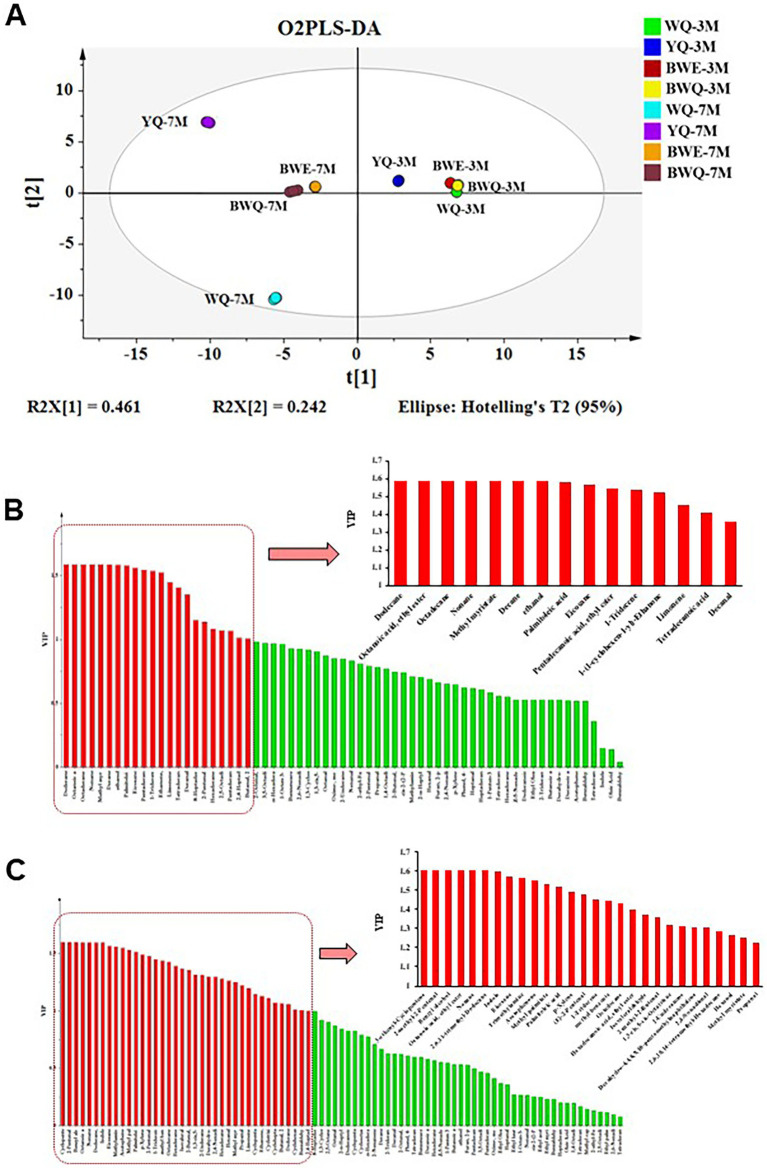
Detection of the differential flavor substances among WQ, YQ, BWQ, and BWE fish sauces at early (3 M) and late (7 M) fermentation. **(A)** O2PLS-DA model. **(B)** Major differential compounds among four fish sauces in the early fermented stage by VIP values. **(C)** Major differential compounds among four fish sauces in the late fermented stage by VIP values.

As shown in Figure 1A, O2PLS-DA model was applied in identifying the potential differential flavor substances among WQ, YQ, BWE and BWQ groups at 3 and 7 M fermentation. A classification model for distinguishing among the eight samples [R^2^(X) = 83.3%, R^2^(Y) = 99.7%, and Q^2^ (*cum*) = 95%] was constructed. The four fish sauces in those two fermentation stages could also be separated well with the average prediction ability of 95%. Moreover, VIP values were utilized to forecast the significance of each variable in the projection, and the main volatile components (VIP >1.2) were selected as differential marker candidates for different fermented fish sauces. This approach has been extensively applied in the screening of vital metabolites in shrimp paste (34) and the detection of potential volatile markers in fermented common carp (7). There were 15 flavors identified as the major differential compounds among four fish sauces in the early fermented stage (Figure 1B). Among them, dodecane, ethyl caprylate, octadecane, nonane, methyl myristate, decane and ethanol were only detected in WQ samples, while palmitoleic acid, eicosane, pentadecanoic acid ethyl ester, 1-tridecene, limonene and decanal were gradually produced in other different rapid fermentation processes. 8-Heptadecene and tetradecanoic acid were not found in the WQ samples, but their contents increased remarkably in other three rapid fermentation processes. Additionally, 28 flavor compounds were deemed as the key difference constituents among different fish sauce samples at the late fermentation stage (Figure 1C). Among them, 3-ethenyl-cyclopentene, 2-methyl-2-pentenal, benzyl alcohol, octanoic acid ethyl ester, nonane and 2,6,11-trimethyldodecane were only found in the natural fermentation WQ groups. The contents of other flavor compounds in YQ, BWE and BWQ groups were significantly increased. Propanal and methyl myristate were not detected in the WQ samples, but their contents increased in other three groups. Therefore, combined with the GC–MS results, 3-ethenyl-cyclopentene, 2-methyl-2-pentenal, benzyl alcohol, octanoic acid ethyl ester, nonane and 2,6,11-trimethyldodecane could be proved to be the potential markers for flavor quality monitoring of the natural fermentation of fish sauce at the late stage, while propanal and methyl myristate were regarded as the key indexes in the rapid fermentation of fish sauces.

### 3.3. Metagenomic sequencing and KEGG database functional annotation of fish sauces

**Figure 2:**
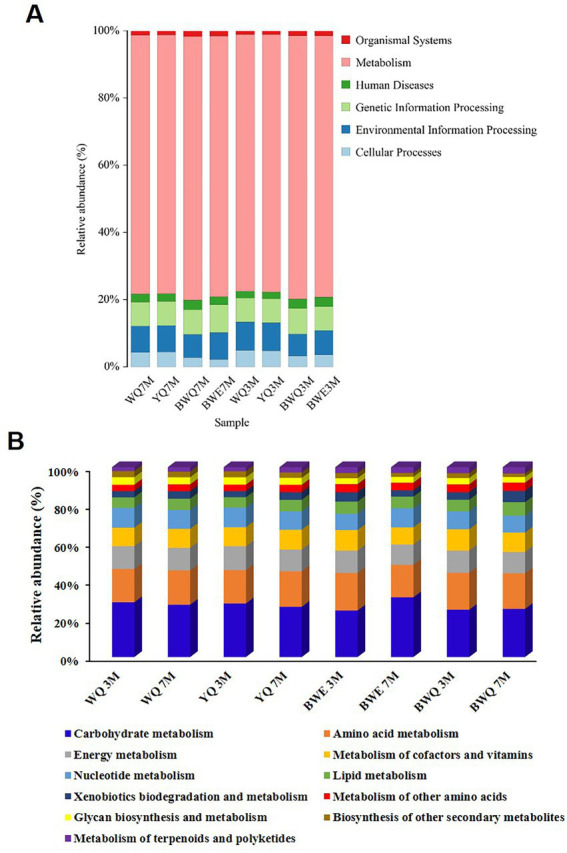
**(A)** Relative abundance of KEGG pathway (at level 1) in different fish sauces. **(B)** Relative abundance of KEGG metabolic pathways (level 2) in different fish sauces.

The commonly assembled metagenomic sequences are depicted in Supplementary Table S1. Figure 2A exhibits a histogram of the relative abundance of eight fermentation samples based on the first-level annotation of the KEGG database. It could be seen that the number of genes belonging to the metabolism process was the largest in all samples, and the relative abundance increased slightly with the progress of fermentation, from the early stage of 76.51–77.58% (3 M of fermentation) to late stage of 76.85–78.77% (7 M of fermentation) in four samples. The relative abundance of genes belonging to genetic information processing and environmental information processing was the second. Additionally, with the progress of BWE and BWQ fermentation, the relative abundance of genes belonging to the classification of human diseases decreased from 0.031 ~ 0.028% to 0.024 ~ 0.022%, which was possibly due to the inhibition of the growth of harmful microorganisms that could cause human diseases during heat preservation fermentation.

Genes belonging to metabolism processes are most directly related to the production of fish sauce flavor substances. Based on the KEGG database, the metabolism functions are further subdivided, and the proportions of different metabolic pathways (level 2) are shown in Figure 2B. The proportion of carbohydrate metabolism among all the samples was the largest, reaching 28.83, 28.22, 24.49, and 24.93% in WQ, YQ, BWE and BWQ groups, respectively, after 3 M of fermentation. With the progress of fermentation, the proportion of carbohydrate metabolism in WQ and YQ groups gradually decreased to 27.55 and 26.44%, while that in BWE and BWQ groups gradually increased to 31.47 and 25.36%, respectively. Proteins, carbohydrates and lipids in raw materials are the main precursors for the formation of flavor compounds. In addition to carbohydrates, flavor formation is also mainly related to amino acid metabolism and lipid metabolism. As can be seen from Figure 2B, compared with 3 M of fermentation, the relative abundance of genes involved in amino acid metabolism in the BWE and BWQ groups decreased after fermentation for 7 M, while the proportions in the WQ and YQ groups increased somewhat, but still accounted for more than 17% during the fermentation process. In contrast, the relative abundance of lipid metabolism genes only changed little.

### 3.4. Key microorganisms related to the formation pathway of characteristic flavor compounds in fish sauce

#### 3.4.1. Carbohydrate metabolism

**Figure 3:**
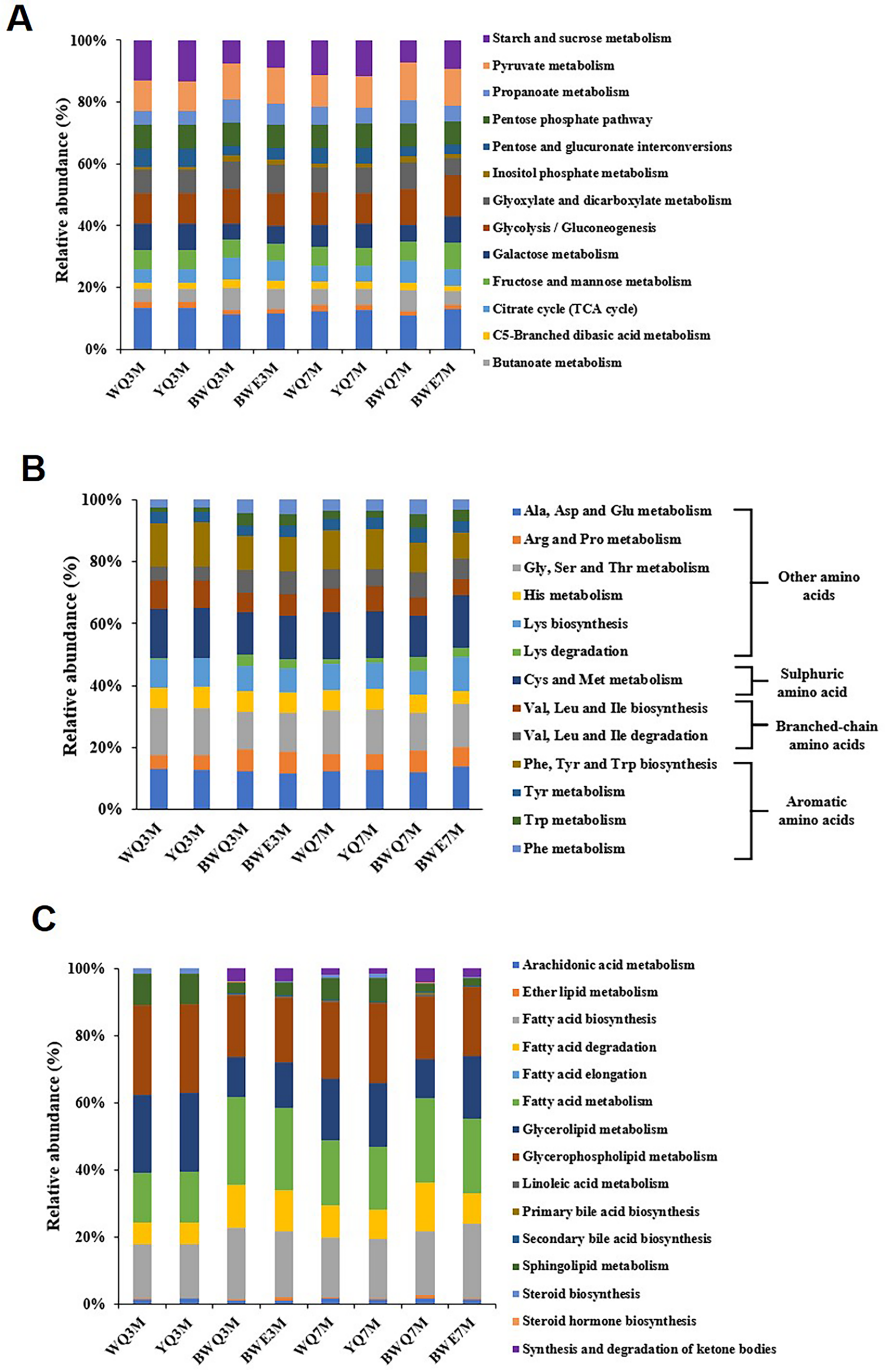
**(A)** Metabolic pathways of carbohydrates for different fish sauces. **(B)** Metabolic pathways of amino acids for different fish sauces. **(C)** Metabolic pathways of lipids for different fish sauces.

The information on the enzymes, microorganisms and the abundance of the genes associated with flavor generation are presented in Table 2. Among carbohydrate metabolism pathways, the number of genes annotated to the glycolysis/gluconeogenesis pathway was the most abundant (Figure 3A). Pyruvate and acetyl-CoA are vital intermediates of carbohydrate metabolism pathway in the formation of flavor. Among them, pyruvate was primarily generated through glycolysis, and the first step was catalyzed by hexokinase (EC 2.7.1.1), which was annotated to three genera in four fish sauces, including *Aspergillus, Halococcus, Virgibacillus*. Glucose-6-phosphate isomerase (EC 5.3.1.9) was a dimeric enzyme which could catalysis the interconversion of D-glucose-6-phosphate and D-fructose-6-phosphate. *Achromobacter, Mycobacterium, Vagococcus, Lactobacillus, Synechococcus, Enterobacter, Stenotrophomonas*, and *Halobacterium* annotated this enzyme. Pyruvate kinase (EC 2.7.1.40) and phosphofructokinase (EC 2.7.1.11/56) were the crucial rate-limiting enzymes in glycolysis (13). The results of annotation indicated that numerous microorganisms in fish sauce were involved in the glycolysis pathway, and *Paenibacillus, Tetragenococcus, Halanaerobium* and *Synechococcus* annotated both two enzymes. Therefore, the pyruvate production in all four fermented fish sauces might need the combined participation of these microorganisms. The changes in the activities of hexokinase, phosphofructokinase and pyruvate kinase in the glycolysis pathway were also obtained in four fish sauces (Supplementary Figure S1).

**Table 2:** Flavor metabolism-related enzymes, gene abundance and microbial distribution of fish sauces.

Flavor forming pathway	KO	Enzyme	KO function	The abundance of the genes				Taxonomy
				WQ	YQ	BWQ	BWE	
Formation of pyruvate	K00844	2.7.1.1	Hexokinase	3	5	8	48	*Aspergillus; Halococcus; Virgibacillus*
	K01810	5.3.1.9	Glucose-6-phosphate isomerase	1,188	2,143	319	620	*Achromobacter; Mycobacterium; Vagococcus; Lactobacillus; Synechococcus; Enterobacter; Stenotrophomonas; Halobacterium*
	K00873	2.7.1.40	Pyruvate kinase	2,155	2,233	387	589	*Staphylococcus; Sphingomonas; Paenibacillus; Leclercia; Tetragenococcus; Halanaerobium; Synechococcus; Pseudoalteromonas; Halobacterium; Immundisolibacter; Stenotrophomonas*
	K00850	2.7.1.11/56	Phosphofructokinase	2,703	2,269	174	479	*Paenibacillus; Aspergillus; Vagococcus; Enterobacter; Halanaerobium; Lactobacillus; Tetragenococcus; Synechococcus*
Formation of acetyl-CoA	K00162	1.2.4.1	Pyruvate dehydrogenase	23	369	419	1,306	*Microbacterium; Synechococcus; Pseudomonas; Acinetobacter; Paenibacillu; Tetragenococcus; Vagococcus; Thalassobius; Achromobacter; Aspergillus; Stenotrophomonas*
	K00627	2.3.1.12	Pyruvate dehydrogenase E2 component	26	181	295	841	*Acinetobacter; Synechococcus; Enterobacter; Pseudomonas; Microbacterium; Tetragenococcus*
	K00656	2.3.1.54	Formate acetyltransferase	882	2,234	46	147	*Vagococcus; Halanaerobium; Tetragenococcus; Paenibacillus; Enterobacter; Peptostreptococcus; Leclercia; Morganella; Escherichia*
	K03737	1.2.7.1	Pyruvate:ferredoxin oxidoreductase	425	0	83	49	*Enterobacter; Vagococcus; Halanaerobium; Leclercia; Synechococcus; Peptostreptococcus*
	K01568	4.1.1.1	Pyruvate decarboxylase	0	0	2	48	*Aspergillus*
	K00156	1.2.5.1	Pyruvate dehydrogenase (quinone)	1	0	15	289	*Enterobacter; Microbacterium; Achromobacter; Acinetobacter; Acidovorax; Stenotrophomonas; Pseudomonas*
	K00128	1.2.1.3	Aldehyde dehydrogenase (NAD+)	6	235	326	816	*Psychrobacter; Mycobacterium; Brevundimonas; Synechococcus; Chroococcidiopsis; Thalassobius*
	K00129	1.2.1.5	Aldehyde dehydrogenase (NAD+)	0	2	0	38	*Aspergillus; Fusarium*
	K01895	6.2.1.1	Acetyl-CoA synthetase	6	225	281	810	*Achromobacter; Synechococcus; Halobacterium; Pseudomonas; Pseudoalteromonas; Enterobacter; Acinetobacter; Brevundimonas*
	K00925	2.7.2.1	Acetate kinase	2,430	2,274	186	498	*Acinetobacter; Synechococcus; Paenibacillus; Psychrobacter; Achromobacter; Vagococcus; Tetragenococcus; Halanaerobium; Lactobacillus; Kushneria; Mycobacterium; Flavobacterium; Microbacterium; Bacteroides; Candidatus; Enterobacter; Pseudomonas*
	K15024	2.3.1.8	Phosphotransacetylase	1,998	2,270	161	361	*Paenibacillus*
	K00132	1.2.1.10	Acetaldehyde dehydrogenase	0	9	5	81	*Enterococcus; Acinetobacter; Idiomarina; Mycobacterium; Psychrobacter; Serratia; Microbacterium*
Formation of lactic acid	K00016	1.1.1.27	L-lactate dehydrogenase	1,681	2,348	137	371	*Lactobacillus; Paenibacillus; Escherichia; Vagococcus; Tetragenococcus; Achromobacter; Halanaerobium; Abyssicoccus; Microbacterium; Leclercia*
	K03778	1.1.1.28	D-lactate dehydrogenase	4	4	57	122	*Lactobacillus; Leclercia; Pseudomonas; Flavobacterium; Peptostreptococcus; Kushneria; Aspergillus; Stenotrophomonas; Staphylococcus*
	K00102	1.1.2.4	D-lactate dehydrogenase (cytochrome)	3	7	25	110	*Achromobacter; Acetobacter; Psychrobacter; Aspergillus; Sphingomonas; Thalassobius*
Formation of acetaldehyde	K01568	4.1.1.1	Pyruvate decarboxylase	0	0	2	48	*Aspergillus*
Formation of ethanol	K18369	1.1.1.1	Alcohol dehydrogenase	2,399	2,181	99	746	*Microbacterium; Aspergillus; Caballeronia*
Formation of acetic acid	K00156	1.2.5.1	Pyruvate dehydrogenase (quinone)	1	0	15	289	*Enterobacter; Microbacterium; Achromobacter; Acinetobacter; Acidovorax; Sphingomonas; Stenotrophomonas; Pseudomonas*
	K00129	1.2.1.5	Aldehyde dehydrogenase (NAD+)	0	2	0	38	*Aspergillus; Fusarium*
	K00128	1.2.1.3	Aldehyde dehydrogenase (NAD+)	6	235	326	816	*Psychrobacter; Mycobacterium; Brevundimonas; Synechococcus; Chroococcidiopsis; Thalassobius*
	K01026	2.8.3.1	Propionate CoA-transferase	0	0	5	20	*Peptostreptococcus; Aureimonas*
	K01067	3.1.2.1	Acetyl-CoA hydrolase	0	0	0	32	*Aspergillus*
	K18118	2.8.3.18	Succinyl-CoA:acetate CoA-transferase	3	0	99	245	*Psychrobacter; Acinetobacter; Halobacterium; Achromobacter; Stenotrophomonas; Agrobacterium; Pseudomonas; Halanaerobium; Pseudoalteromonas; Brevundimonas; Sphingomonas*
Formation of acetoin	K11258	4.1.3.18	Acetolactate synthase	11	7	98	120	*Pseudoalteromonas; Stenotrophomonas; Lactobacillus*
	K01575	4.1.1.5	Acetolactate decarboxylase	6	58	28	80	*Enterobacter; Synechococcus; Synechococcus; Tetragenococcus; Lactobacillus; Mycobacterium; Staphylococcus*
Formation of fatty alcohols and aldehydes	K19246	1.13.11	Lipoxygenase	0	0	18	161	*Enterococcus; Aspergillus;(fungi) Staphylococcus*
	K10528	4.1.2.-	Hydroperoxide lyase	19	0	5	6	*Lactobacillus; Tetragenococcus; Staphylococcus*
	K15389	1.3.99.3	General acyl-CoA dehydrogenase	0	0	75	7	*Phialosimplex; Acinetobacter; Escherichia; Mycobacterium; Paracoccus; Aspergillus; Pseudomonas; Pseudoalteromonas; Luteibacter*
	K13767	4.2.1.17	Enoyl-CoA hydratase	1,984	2,208	198	1,276	*Immundisolibacter; Achromobacter; Mycobacterium; Pseudomonas; Thalassobius; Halanaerobium*
	K07516	1.1.1.35	hydroxyacyl-CoA dehydrogenase (HADA)	2	15	67	285	*Halobacterium; Achromobacter; Sphingomonas; Paenibacillus; Stenotrophomonas; Vagococcus; Thalassobius; Flavobacterium; Lentibacillus; Acinetobacter*
	K00626	2.3.1.9	Acetyl-CoA C-acetyltransferase	3	59	266	1,289	*Achromobacter; Mycobacterium; Comamonas; Acinetobacter; Thalassobius; Vagococcus; Mycobacterium; Pseudoalteromonas; Stenotrophomonas; Enterobacter; Phenylobacterium*	
	K00053	1.1.1.86	Ketol-acid reductoisomerase	1,933	3,374	102	507	*Sphingomonas; Synechococcus; Acinetobacter; Psychrobacter; Stenotrophomonas; Pseudoalteromonas; Pseudomonas; Klebsiella; Aspergillus; Enterobacter; Halanaerobium; Paenibacillus; Phenylobacterium*	K01897	6.2.1.3	Long-chain acyl-CoA synthetase	2,013	2,163	373	1,078	*Sphingomonas; Luteibacter; Mycobacterium; Achromobacter; Microbacterium; Psychrobacter; Halanaerobium; Synechococcus; Pseudomonas; Halobacterium; Paenibacillus; Staphylococcus; Bacillus; Oceanobacillus; Virgibacillus; Lentibacillus*
Formation of aromatic aldehydes and alcohols	K01593	4.1.1.28	Aromatic-L-amino acid decarboxylase	0	0	13	24	*Phialosimplex; Pseudomonas; Aspergillus*
	K00274	1.4.3.4	Monoamine oxidase	2	110	64	418	*Pseudomonas; Brevibacillus; Synechococcus; Psychrobacter; Microbacterium*
	K00055	1.1.1.90	Aryl-alcohol dehydrogenase	0	0	24	23	*Stenotrophomonas; Psychrobacter; Acinetobacter*
	K00146	1.2.1.39	Phenylacetaldehyde dehydrogenase	0	0	10	40	*Pseudomonas; Acinetobacter; Achromobacter*
	K00832	2.6.1.57	Aromatic-amino acid transaminase	0	8	43	221	*Psychrobacter; Achromobacter; Stenotrophomonas; Acinetobacter; Agrobacterium; Pseudomonas; Enterobacter; Phenylobacterium*
	K00167	1.2.4.4	α-keto acid decarboxylases	0	36	71	408	*Pseudoalteromonas; Pseudomonas; Achromobacter; Paenibacillus; Vagococcus; Sphingomonas; Shewanella; Brevundimonas; Aspergillus; Flavobacterium*
	K00276	1.4.3.21	Primary-amine oxidase	0	3	25	237	*Microbacterium; Acinetobacter; Aspergillus*
	K00285	1.4.5.1	D-amino acid dehydrogenase	0	33	73	452	*Achromobacter; Pseudomonas; Stenotrophomonas; Acinetobacter; Enterobacter*
	K00824	2.6.1.21	Alanine transaminase	0	12	38	49	*Achromobacter; Paenibacillus; Aspergillus; Mycobacteroides*
	K01754	4.3.1.19	Threonine deaminase	3	158	198	728	*Achromobacter; Synechococcus; Pseudomonas; Mycobacterium; Paenibacillus; Pseudoalteromonas*
	K00826	2.6.1.42	Branched-chain amino acid aminotransferase	1741	2097	208	671	*Achromobacter; Phialosimplex; Synechococcus; Paenibacillus; Microbacterium; Stenotrophomonas; Pseudomonas; Halobacterium; Halanaerobium*
Formation of fatty acids	K11987	1.14.19.1	Stearoyl-CoA desaturase	6	478	183	538	*Stenotrophomonas; Synechococcus; Achromobacter; Acinetobacter; Stenotrophomonas*
	K00508	1.14.19.3	Linoleoyl-CoA desaturase	0	0	13	256	*Acinetobacter; Stenotrophomonas; Mycobacterium; Pseudomonas*
	K01961	6.4.1.2	Acetyl-CoA carboxylase	8	275	199	717	*Virgibacillus; Halococcus; Staphylococcus; Oceanobacillus; Morganella; Vibrio*
	K11533	2.3.1-	Fatty acid synthase	3,360	4,167	314	1,502	*Mycobacterium; Williamsia*
	K01071	3.1.2.21	Medium-chain acyl-hydrolase	0	0	11	16	*Vagococcus; Lactobacillus; Tetragenococcus*
Formation of esters	K03928	3.1.1.1	Carboxylesterase	1,609	2,244	28	339	*Synechococcus; Achromobacter; Vagococcus; Stenotrophomonas; Acinetobacter; Halanaerobium; Tetragenococcus*
	K01046	3.1.1.3	Triacylglycerol lipase lipase ATG15	14	76	144	173	*Synechococcus; Acinetobacter; Mycobacterium; Pseudomonas; Aspergillus*
	K18369	1.1.1.1	Alcohol dehydrogenase	2,399	2,181	99	746	*Microbacterium; Aspergillus; Caballeronia*

The citrate cycle/TCA pathway was also the main pathway of carbohydrate metabolism in fish sauce. The relative abundance of the citrate cycle pathway decreased gradually with fish sauce fermentation, from 7.02% at the early stage to 5.58% at the 7 months of fermentation. The acetyl-CoA is the beginning intermediate of the citrate cycle and the key precursor of fatty acids, amino acids and polyketones. Generally, four formation pathways of acetyl-CoA were detected in fish sauces. First, the pyruvate dehydrogenase (EC 1.2.4.1, 2.3.1.12) could be able to catalyze the direct transformation of pyruvate to acetyl-CoA (7). *Tetragenococcus*, *Paenibacillu*, *Vagococcus*, *Achromobacter*, *Enterobacter*, *Microbacterium*, *Pseudomonas*, *Synechococcus*, *Acinetobacter*, *Thalassobius*, *Aspergillus*, and *Stenotrophomonas* annotated this enzyme in all four fish sauces. Second, in numerous prokaryotes and several anaerobic eukaryotes, pyruvate could also be converted to acetyl-CoA through formate acetyltransferase (EC 2.3.1.54) or pyruvate: ferredoxin oxidoreductase (EC 1.2.7.1) (35). EC 2.3.1.54 was mostly annotated to *Vagococcus, Halanaerobium, Tetragenococcus, Paenibacillus, Enterobacter, Peptostreptococcus, Leclercia, Morganella*, and *Escherichia*, while EC 1.2.7.1 was primarily annotated to *Halanaerobium, Leclercia, Vagococcus, Synechococcus, Enterobacter*, and *Peptostreptococcus*. Based on the results of our previous research on microbial abundance, we found that even a low abundance of microorganisms could contribute to fermentation. The third pathway was to first convert pyruvate into acetaldehyde through pyruvate decarboxylase (EC 4.1.1.1, 1.2.5.1), and then acetaldehyde dehydrogenase (EC 1.2.1.3, 1.2.1.5) converted the acetaldehyde to acetate, followed by synthesizing to acetyl-CoA through acetyl-CoA synthase (EC 6.2.1.1). The entire pathway needed the joint participation of various microorganisms in fermented fish sauces, as shown in Table 2. Moreover, acetate could be converted to acetyl phosphate *via* acetate kinase (EC 2.7.2.1), followed by forming acetyl-CoA through phosphotransacetylase (EC 2.3.1.8). *Paenibacillus* mainly annotated this pathway. Additionally, unlike the above pathway, the fourth pathway was the direct conversion of acetaldehyde to acetyl-CoA through acetaldehyde dehydrogenase (EC 1.2.1.10), and seven bacterial genera were able to participate in this pathway in fish sauces. In general, pyruvate was presumably converted to acetyl-CoA by the *Halococcus*, *Lactobacillus*, *Halobacterium*, and *Aspergillus* in fish sauce fermentation through the glycolysis pathway.

As the main fermentation end product, the generation of lactic acid primarily came from the reduction of pyruvate. D-lactate dehydrogenase (EC 1.1.1.28) and L-lactate dehydrogenase (EC 1.1.1.27) catalyzed the conversion of pyruvate and lactate under anoxic conditions. Another lactate dehydrogenase (EC 1.1.2.4) could produce pyruvate in the reverse direction by consuming lactic acid. Table 2 demonstrated that *Lactobacillus*, *Paenibacillus*, *Tetragenococcus* and *Staphylococcus* were the key microorganisms that accumulated lactic acid in fish sauce, and *Leclercia* also facilitated the generation of lactic acid. However, *Achromobacter*, *Acetobacter*, and *Aspergillus* were primarily annotated to the consumption of lactic acid. At certain levels of lactic acids, the capacity of microbes to utilize lactic acid was conducive to survival. In addition, ethanol, acetic acid and acetoin were also the main products of the glycolysis pathway in fish sauce. For instance, ethanol was produced through glycolysis pathway and was further converted by acetaldehyde through ethanol dehydrogenase (EC 1.1.1.1) (36). *Microbacterium*, *Aspergillus*, and *Caballeronia* were mainly involved in the transformation of acetaldehyde to ethanol in four fish sauces fermentation. There were seven catalytic enzymes associated with acetic acid production in fish sauces by sequencing analysis. The enzymes participated in the production of acetic acid from pyruvate were pyruvate dehydrogenase (EC 1.2.5.1), which was annotated to *Enterobacter*, *Microbacterium*, *Pseudomonas*, etc. Propionate CoA-transferase (EC 2.8.3.1), acetyl-CoA hydrolase (EC 3.1.2.1), succinyl-CoA: acetate CoA-transferase (EC 2.8.3.18), and acetate-CoA ligase (EC 6.2.1.13) were all involved in the conversion of acetyl-CoA to acetic acid. However, only the first three enzymes were annotated in BWQ and BWE samples. Thus, it was hard to generate acetic acid through this pathway in the fermentation process of fish sauce. Moreover, acetic acid could be formed through the catalysis of acetaldehyde under the action of acetaldehyde dehydrogenase (EC 1.2.1.5, EC 1.2.1.3, EC 1.2.5.2, and EC 1.2.1). However, only the first two enzymes were detected in four fish sauces, and a total of 8 genera were annotated, including *Aspergillus*, *Psychrobacter*, *Mycobacterium*, *Synechococcus*, etc. For acetoin, pyruvate was usually regarded as the precursor. Results indicated that acetolactate synthase (EC 4.1.3.18) was annotated to *Lactobacillus*, *Pseudoalteromonas* and *Stenotrophomonas*, which could catalyze the formation of two molecules of pyruvate into acetolactic acid. Then, acetolactic acid was synthesized into acetoin through α-acetolactic decarboxylase (EC 4.1.1.5). In all the samples, *Synechococcus*, *Tetragenococcus*, *Lactobacillus*, *Mycobacterium*, and *Staphylococcus* were the major underlying acetoin-producing bacteria. Notably, *Lactobacillus* could annotate the complete formation pathway of acetoin. Our results were similar to Andrea et al. who reported that *Lactobacillus crustorum* LMG 23699 included all the enzyme coding genes that converted citric acid into acetoin, lactic acid, acetic acid and others (37). Additionally, since fish meat is mainly rich in protein and amino acids, but low in sugar content, the gluconeogenesis pathway may be a sign of the transformation of amino acids to glucose during the fermentation of fish sauce.

#### 3.4.2. Amino acid metabolism

Protein degradation and amino acid metabolism are one of the most critical ways for the flavor formation of fermented foods such as fish sauce. The amino acid metabolism pathway annotated by KEGG is shown in Figure 3B. The flavor precursor amino acids were mainly aromatic amino acids (Phe, Tyr, and Trp), branched-chain amino acids (Leu, Ile and Vas), and sulphuric amino acid (Cys and Met). Among all the amino acid metabolism pathways noted in four fish sauces, the proportion of aromatic amino acid metabolism was the highest (19.17 ~ 23.64%), followed by sulphuric amino acid (13.54 ~ 16.73%) and branched-chain amino acid (11.81 ~ 13.87%). In the fermentation process of fish sauce, the relative abundance of Phe, Tyr and Trp metabolism decreased gradually in all the fish sauces except for YQ group. Phe was usually the main precursor of phenylacetaldehyde, phenylacetic acid, phenyllactic acid, styrene and phenylethanol, while phenols mainly came from the degradation of Tyr. Moreover, the relative abundance of Val, Leu and Ile degradation pathways also decreased during fermentation, and those branched-chain amino acids could be reduced to isobutanol, 2-methyl-butanal and 2-methyl-butanol after transamination and decarboxylation.

The flavors of fish sauce such as phenylethanol, 2-methyl-butanal, benzaldehyde and benzyl alcohol, belonged to higher alcoholaldehydes, which largely came from amino acid metabolism. Currently, two pathways have been reported for the generation of phenylethanol (Table 2). Phe was first converted to phenylethylamine through the aromatic-L-amino acid decarboxylase (EC 4.1.1.28). After removing the amine group by monoamine oxidase (EC 1.4.3.4), phenylethylamine was transferred into phenylacetaldehyde, followed by converting to phenylethanol through phenylacetaldehyde dehydrogenase (EC 1.2.1.39) or aryl-alcohol dehydrogenase (EC 1.1.1.90) (38). However, EC 4.1.1.28, EC 1.2.1.39 and EC 1.1.1.90 were only annotated in BWE and BWQ groups. Alternatively, Phe was converted to phenylpyruvate and phenylacetaldehyde through aromatic-amino acid transferase (EC 2.6.1.57) and α-keto acid decarboxylase (EC 1.2.4.4), followed by reducing to phenylethanol. Among them, EC 2.6.1.57 was primarily annotated to *Achromobacter*, *Stenotrophomonas*, *Acinetobacter*, *Agrobacterium*, *Pseudomonas*, *Enterobacter*, and *Phenylobacterium*. Meanwhile, EC 1.2.4.4 was annotated to *Pseudoalteromonas*, *Pseudomonas*, *Achromobacter*, *Paenibacillus*, *Vagococcus*, *Sphingomonas*, *Shewanella*, *Brevundimonas*, *Aspergillus*, and *Flavobacterium* in YQ, BWQ and BWE samples. *Pseudomonas* could be involved in the complete formation of phenylacetaldehyde. Furthermore, *Stenotrophomonas*, *Psychrobacter*, *Pseudomonas*, *Acinetobacter*, and *Achromobacter* could involve in the conversion of phenylacetaldehyde to phenylethanol in both BWQ and BWE groups. Moreover, amino acid oxidase (EC 1.4.3.21), alanine transaminase (EC 2.6.1.2/21) and D-amino acid dehydrogenase (EC 1.4.5.1) also catalyzed the formation of corresponding α-ketoacid from Phe. Other α-ketoacid could also be generated by a chain growth reaction of α-ketyl butyric acid originating from threonine (Thr) deamination catalyzed by Thr deaminase (EC 4.3.1.19). Therefore, phenylacetaldehyde might be derived from the conversion of Phe under the action of α-ketoacid decarboxylase and aromatic amino acid transferase. Benzaldehyde could also be generated from Phe as a precursor. Briefly, Phe was transferred into cinnamic acid through Phe ammonia-lyase, followed by generating benzaldehyde under the action of benzaldehyde synthase (27). Nevertheless, this was only a theoretical method and required to be verified experimentally.

2-Methyl-butanal was formed using Leu or Ile as the precursor. Branch-chain amino acid transaminases (EC 2.6.1.42) that catalyzed Leu into α-ketoacid were annotated to 9 genera. Ten genera were annotated to α-ketoacid decarboxylase (EC 1.2.4.4). EC 1.2.4.4 and EC 2.6.1.42 were both annotated to *Paenibacillus*, *Achromobacter*, and *Pseudomonas*, exhibiting the significance of these genera in the generation of 2-methyl-butanal. It has been manifested that *Paenibacillus* was the intrinsic bacteria promoting flavor formation in traditionally fermented fish (39). Furthermore, the intermediates from citric acid metabolism could also participate in the amino acid metabolism pathway to synthesize amino acids or various flavor substances.

#### 3.4.3. Lipid metabolism

The metabolic pathway of lipids is another key pathway for flavor formation. In the lipid metabolism diagram annotated by KEGG (Figure 3C), glycerolipid metabolism and glycerophospholipid metabolism accounted for a large proportion. It was found that glycerophospholipids and glycerolipids could be degraded to fatty acids under the action of lipase, and the free fatty acids of fish sauce primarily came from the degradation of glycerophospholipids (40). Additionally, the biosynthesis of fatty acids gradually increased with the fermentation of fish sauce, and the relative abundance in WQ, YQ, BWQ and BWE samples at 7 M of fermentation reached 17.53, 17.80, 19.06, and 22.38%, respectively. And the changing trend of the biosynthetic pathway of unsaturated fatty acids was consistent with that of total fatty acids, which showed that microorganisms during the fermentation process of fish sauce had the ability to synthesize fatty acids. Moreover, the relative abundance of fatty acid degradation pathway accounted for 6.51 ~ 14.33% in all samples, which might be related to the formation of branched fatty aldehydes, ketones, alcohols and esters.

The characteristic flavor of fish sauce including decanal, hexanal, octanal, nonanal, 2,4-heptadienal and 2,6-nonadienal belonged to fatty aldehydes. The vital enzymes that participated in the flavor formation pathway vary with different substrates (Table 2). Among them, lipoxygenase (EC 1.13.11) and hydroperoxide lyase (EC 4.1.2.-) were the key enzymes of the lipoxygenase pathway. For example, hexanal and 2,6-nonadienal were catalyzed by lipoxygenase and hydroperoxide lyase when linolenic acid was used as the precursor. Nevertheless, decanal, octanal, nonanal and (E,Z)-2,6-nondienal were produced since lipoxygenase of *Pyropia haitanensis* was incubated with the hydrolyzed fish oil (41). The pathway of β-oxidation was also in charge of the generation of secondary alcohols, methyl ketones and aldehydes. Several enzymes, such as general acyl-CoA dehydrogenase (EC 1.3.99.3), enoyl-CoA hydratase (EC 4.2.1.17), hydroxyacyl-CoA dehydrogenase (EC 1.1.1.35), acetyl-CoA C-acetyltransferase (EC 2.3.1.9) and ketol-acid reductoisomerase (EC 1.1.1.86) were involved in this pathway. The sequencing results of fish sauce could annotate the whole lipoxygenase and β-oxidation pathway. Among them, *Staphylococcus* was able to participate in the lipoxygenase pathways, while *Halanaerobium* and *Mycobacterium* were involved in the whole β-oxidation pathway. Moreover, acyl-CoA might be generated by long-chain acyl-CoA synthetase (EC 6.2.1.3) since FFA were precursors, followed by converting to fatty aldehydes under the action of acyl-CoA reductase (EC 1.2.1.84), while EC 1.2.1.84 was not annotated to any microorganisms in all the fish sauces.

The biosynthesis of esters is normally executed by alcoholysis and esterification. Generally, esters could be classified into two types: acetate ester and FFA ethyl ester. The main acetate esters in fish sauce were ethyl acetate, while ethyl decanoate and ethyl caprylate in fish sauce were FFA ethyl esters. In our study, three pathways catalyzed the esters synthesis. First, carboxylesterases (EC 3.1.1.1) and triacylglycerol lipase (EC 3.1.1.3) belonging to the pathway of ethanol to ethyl ester were annotated to 11 genera, in which alcohol dehydrogenase (EC 1.1.1.1) could typically reduce ketones or aldehydes to ethanol, and oxidize hemiacetal compounds to form esters. The microorganisms that were annotated to EC 1.1.1.1 included *Microbacterium*, *Aspergillus* and *Caballeronia* in fish sauce samples. Second, acetate ester was generally produced by the esterification of acyl-CoA with alcohols through alcohol O-acetyltransferase (EC 2.3.1.84), but this enzyme was not annotated to any microorganism. Moreover, one of the precursors of FFA ethyl ester was fatty acid, so the formation pathway of fatty acid was also related to the formation of ester. The precursor fatty acids of ethyl decanoate and ethyl caprylate are octanoic acid and decanoic acid, respectively. The fatty acids in fish sauce were mainly long-chain fatty acids (LCFAs), most of which came from the hydrolysis of lipids, but might also come from microbial synthesis. Fatty acid desaturases, such as stearoyl-CoA desaturase (EC 1.14.99.1) and linoleoyl-CoA desaturase (EC 1.14.19.3), were the key enzyme for the synthesis of LCFAs, while the short-chain fatty acids (SCFAs) might come from the hydrolysis of LCFAs or the synthesis of microorganisms. The precursor of fatty acid synthesis was acetyl-CoA, which could be synthesized *de novo*, or formed by carbon chain extension based on existing fatty acids (42). For example, acetyl-CoA carboxylase (EC 6.4.1.2) was utilized to transfer acetyl-CoA into malonyl-CoA, which was annotated to 6 bacterial genera in fish sauce. Then, the fatty acid synthase (EC 2.3.1-) involved in the carbon chain extension reaction could synthesize fatty acids through multi-step pathways using malonyl-CoA as the substrate. The results of fish sauce sequencing showed that only *Mycobacterium* and *Williamsia* were annotated in bacterial genera. Finally, fatty acids needed to be released from acyl carrier proteins through medium-chain acyl-hydrolase (EC 3.1.2.21), which was related to *Vagococcus*, *Lactobacillus*, and *Tetragenococcus*.

### 3.5. Metabolic network formed by characteristic flavor of fish sauces

**Figure 4:**
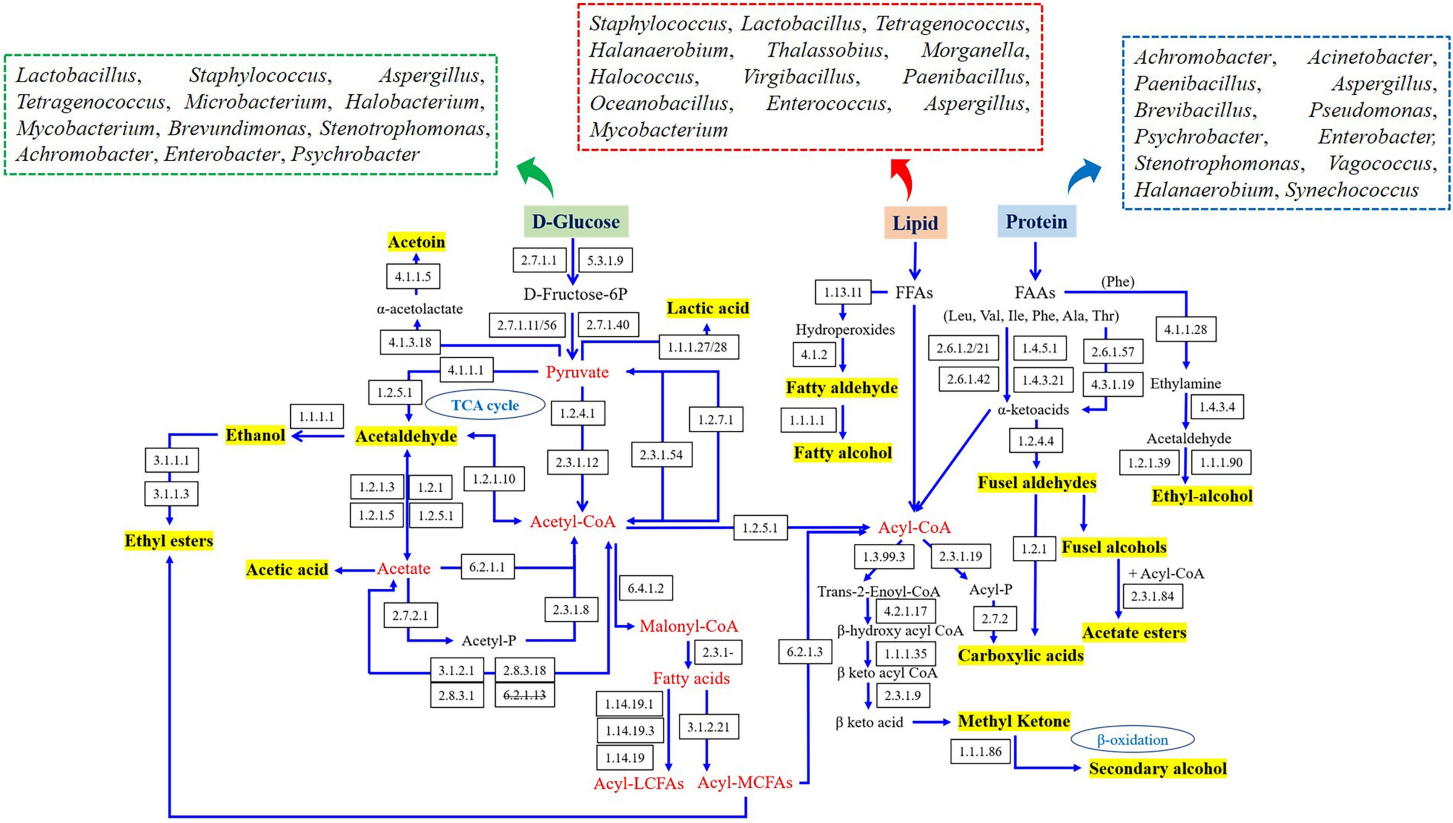
Prediction of the metabolic network and related microorganisms in the dominant flavor formation of fish sauce.

The metabolic networks of characteristic flavor in fish sauce were established according to the literatures and KEGG database, and the related microorganisms in various flavor generation pathways are summarized in Figure 4. The flavors produced by carbohydrate metabolism primarily contained lactic acid, acetaldehyde, ethanol, acetoin and acetic acid, which required the combined participation of *Lactobacillus*, *Staphylococcus*, *Aspergillus*, *Tetragenococcus*, *Microbacterium*, *Halobacterium*, *Mycobacterium*, *Brevundimonas*, *Stenotrophomonas*, *Achromobacter*, *Enterobacter*, and *Psychrobacter*. Flavors originated from the metabolism of amino acids were primarily generated by *Achromobacter*, *Acinetobacter*, *Paenibacillus*, *Aspergillus*, *Brevibacillus*, *Pseudomonas*, *Psychrobacter*, *Stenotrophomonas*, *Halanaerobium*, *Synechococcus*, *Vagococcus*, and *Enterobacter*, including some aromatic aldehydes and alcohols. In addition, flavor derived from lipid metabolism primarily included fatty acids, fatty alcohols, aldehydes and esters, and several bacteria genera such as *Staphylococcus*, *Lactobacillus*, *Tetragenococcus*, *Halanaerobium*, *Thalassobius*, *Morganella*, *Halococcus*, *Virgibacillus*, *Paenibacillus*, *Oceanobacillus*, *Enterococcus*, *Aspergillus*, and *Mycobacterium* had vital effects on those flavor synthesis.

In brief, the top 10 bacteria with the highest number of enzymes associated with flavor generation were *Lactobacillus*, *Staphylococcus*, *Enterobacter*, *Aspergillus*, *Tetragenococcus*, *Halanaerobium*, *Mycobacterium*, *Psychrobacter*, *Stenotrophomonas*, and *Paenibacillus*, which appeared to play a part in the flavor generation of fish sauce. Although there were certain differences in the flavor substances of fish sauce produced by different fermentation methods, the four kinds of fish sauce had similar flavor formation pathways. The difference was that only BWQ and BWE groups could produce ethyl-alcohol through amino acid metabolism, while YQ, BWE and BWQ groups could generate phenylacetaldehyde through the transformation of Phe by α-ketoacid decarboxylase and aromatic amino acid transferase. Meanwhile, only BWE and BWQ samples could produce secondary alcohols, methyl ketones and aldehydes through the complete β-oxidation pathway. Combined with the study of the dynamics of microbial community in genus level (data not shown), *Halanaerobium*, *Tetragenococcus*, *Lactobacillus*, *Staphylococcus*, *Stenotrophomonas*, and *Paenibacillus* were mainly flavored microbe in fish sauce, and the formation of characteristic flavors probably be the consequence of the synergetic actions of various microorganisms. Nevertheless, although a metabolic network connecting key microorganisms that drive the synthesis of flavor was established, the metabolism of every microorganism in the community might change over time as fermentation progressed. Therefore, future research should focus on the dynamic expression and gene regulation by transcriptomic, metabonomic and proteomic.

## 4. Conclusion

This article studied the relationship between the microorganisms and flavor of different fermented fish sauces. 15 and 28 characteristic flavors confirmed to be the major differential compounds among four fish sauces in the early and late fermentation stage. By annotating KEGG pathway of fish sauces, a total of 56 enzymes related to the metabolism of fish sauce flavor components were identified. The formation of fatty aldehydes and alcohols in BWE and BWQ samples was primarily annotated to lipoxygenase and β-oxidation pathways, while that in YQ and WQ groups was only annotated to the complete acetyl-CoA pathway. Furthermore, 10 key microorganisms with different enzyme-coding genes participated in the flavor biosynthesis were screened, which could be further utilized to structure a synthetic microbiota. *Staphylococcus* was involved in the lipoxygenase pathways, while *Halanaerobium* was involved in the complete β-oxidation pathway and the generation of aldehydes and alcohols came from branched amino acids. *Enterococcus*, *Aspergillus*, *Paenibacillus*, and *Stenotrophomonas* could jointly participate in the formation of alcoholaldehydes derived from aromatic amino acids. This study is of great significance to optimize the key microorganisms for improving the flavor of fish sauce.

## Data availability statement

The original contributions presented in the study are included in the article/Supplementary material, further inquiries can be directed to the corresponding author.

## Author contributions

JRH and QG conceived the project idea and obtained fundings. JRH and TK were assigned the project, do the research, and wrote this article. JLJ, XZ, and XLZ finished all the research. PL and QG revised the manuscript. All authors approved the final version of the manuscript.

## Funding

This work was supported financially by the Public Welfare Technology Application Research Project of Zhejiang Province (LGN22C200006), the Chinese Academy of Engineering Academy-Locality Cooperation Project (no. 2019-ZJ-JS-02), and the General Project of Education Department of Zhejiang Province (Y202147299).

## Conflict of interest

The authors declare that the research was conducted in the absence of any commercial or financial relationships that could be construed as a potential conflict of interest.

## Publisher’s note

All claims expressed in this article are solely those of the authors and do not necessarily represent those of their affiliated organizations, or those of the publisher, the editors and the reviewers. Any product that may be evaluated in this article, or claim that may be made by its manufacturer, is not guaranteed or endorsed by the publisher.

## Supplementary material

The Supplementary material for this article can be found online at: https://www.frontiersin.org/articles/10.3389/fnut.2023.1121310/full#supplementary-material
