# Supplementary material for: Characteristic flavor metabolic network of fish sauce microbiota with different fermentation processes based on metagenomics
Source: Front Nutr. 2023 Mar 6;10:1121310. doi: 10.3389/fnut.2023.1121310 (PMC10025566; doi:10.3389/fnut.2023.1121310)
Supplement: Supplementary file 5 [file Data_Sheet_5.pdf]

Table S1. Overview of whole metagenomics sequencing information (7M)

Sample	WQ	YQ	BWQ	BWE
Raw data（Mbp）	10228.4883	10357.26	10648.74	10204.32
Clean data（Mbp）	8654.58285	8620.288	8974.492	8699.703
Number of Reads	29013112.7	29057066	31240950	29778748
GC（%）	34.27	35.55	44.95	44.91
Q20（%）	97.04	97.05333	94.95333	95.74667
Q30（%）	91.7533333	92	89.21333	90.19333
Contig number	49889.3333	113900	970401.7	743635.7
scatigs N50 length（bp）	744	778	572.6667	566.6667

Q20 (%): The percentage of base quality value greater than or equal to 20 or the base number with sequencing error rate less than 1%. Q30 (%): The percentage of base quality value greater than or equal to 30 or the base number with sequencing error rate less than 0.1%

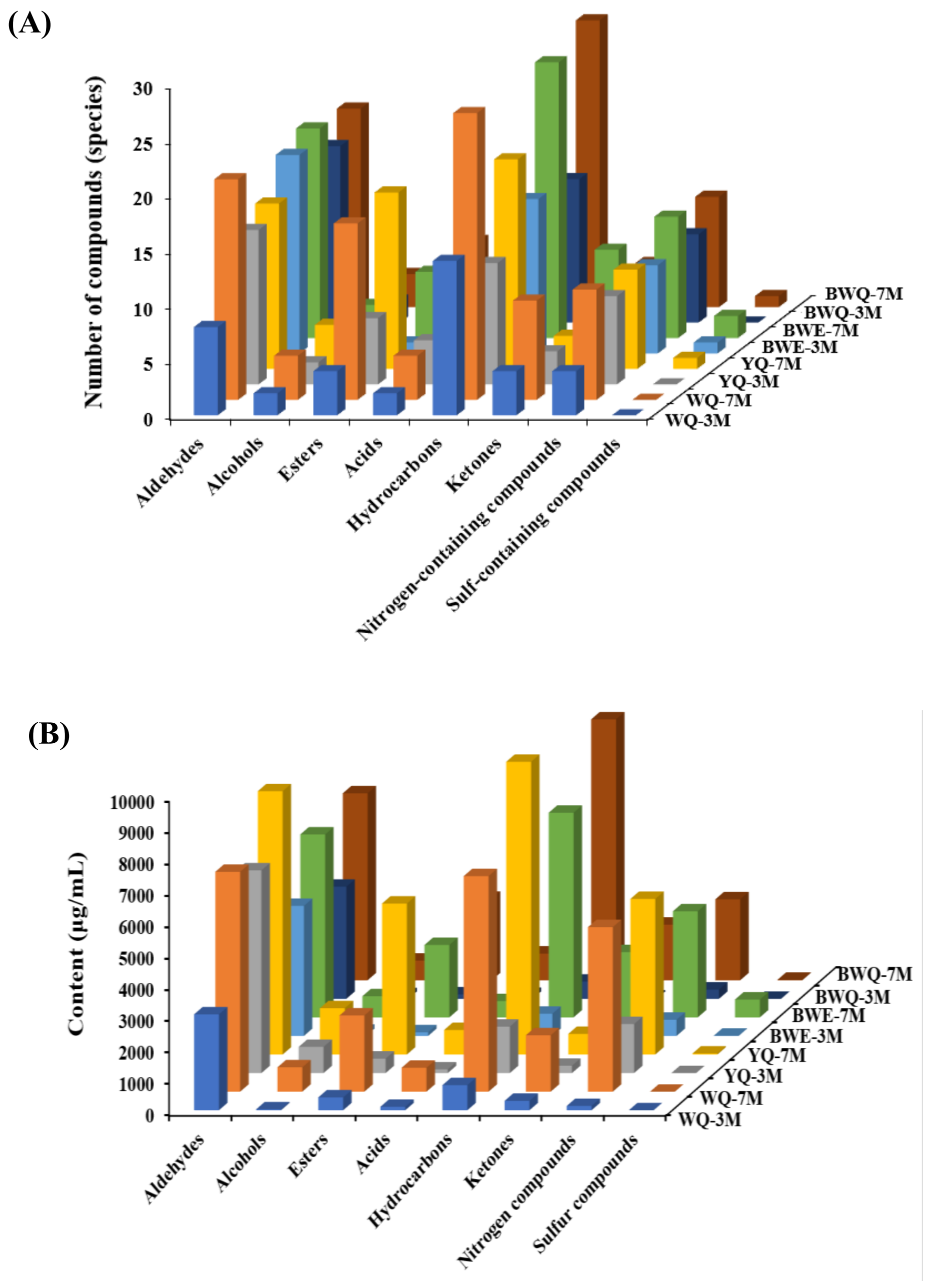

Fig. S1 The amount (A) and total concentration (B) of volatile flavor compounds in different fish sauces

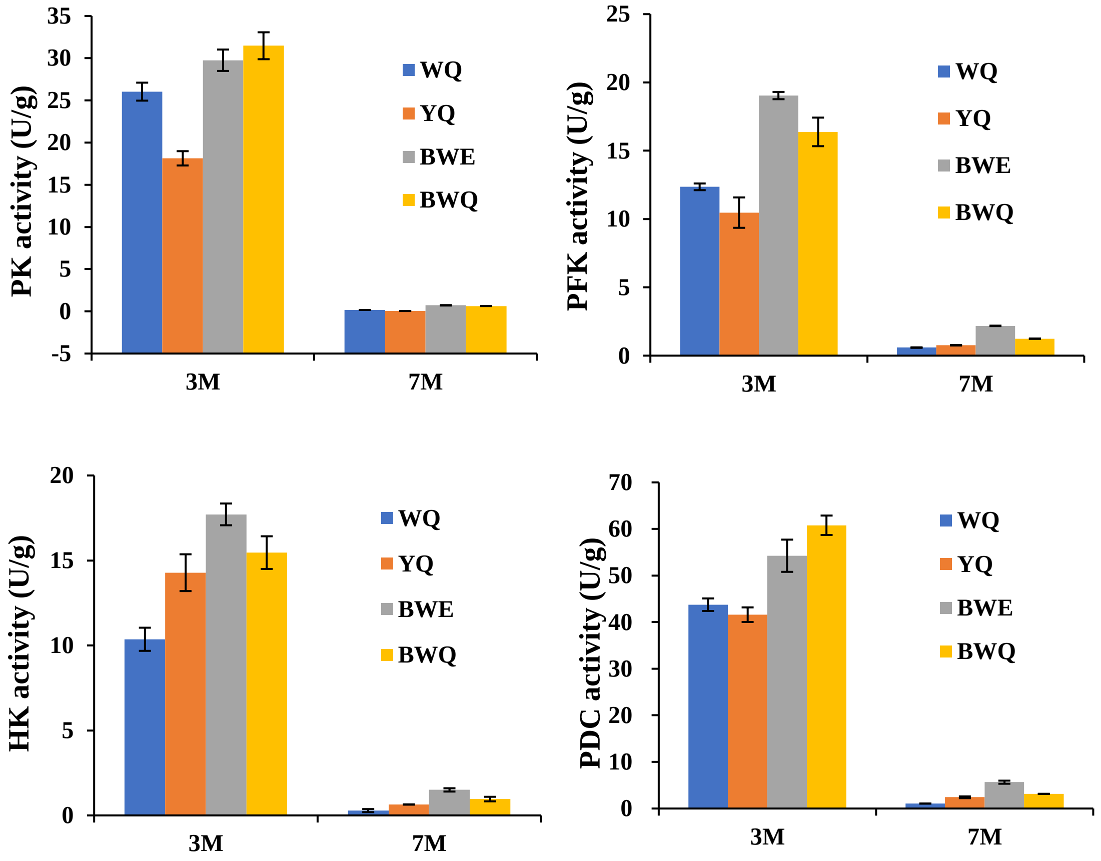

Fig. S2 The changes in the activities of key enzymes HK, PFK and PK in the glycolysis pathway during fish sauce fermentation. HK: hexokinase; PFK: phosphofructokinase; PK: pyruvate kinase; PDC: pyruvate decarboxylase
