# Supplementary figures and images for: Characteristic flavor metabolic network of fish sauce microbiota with different fermentation processes based on metagenomics
Source: Front Nutr. 2023 Mar 6;10:1121310. doi: 10.3389/fnut.2023.1121310 (PMC10025566; doi:10.3389/fnut.2023.1121310)

IF3M1 Length Distribution

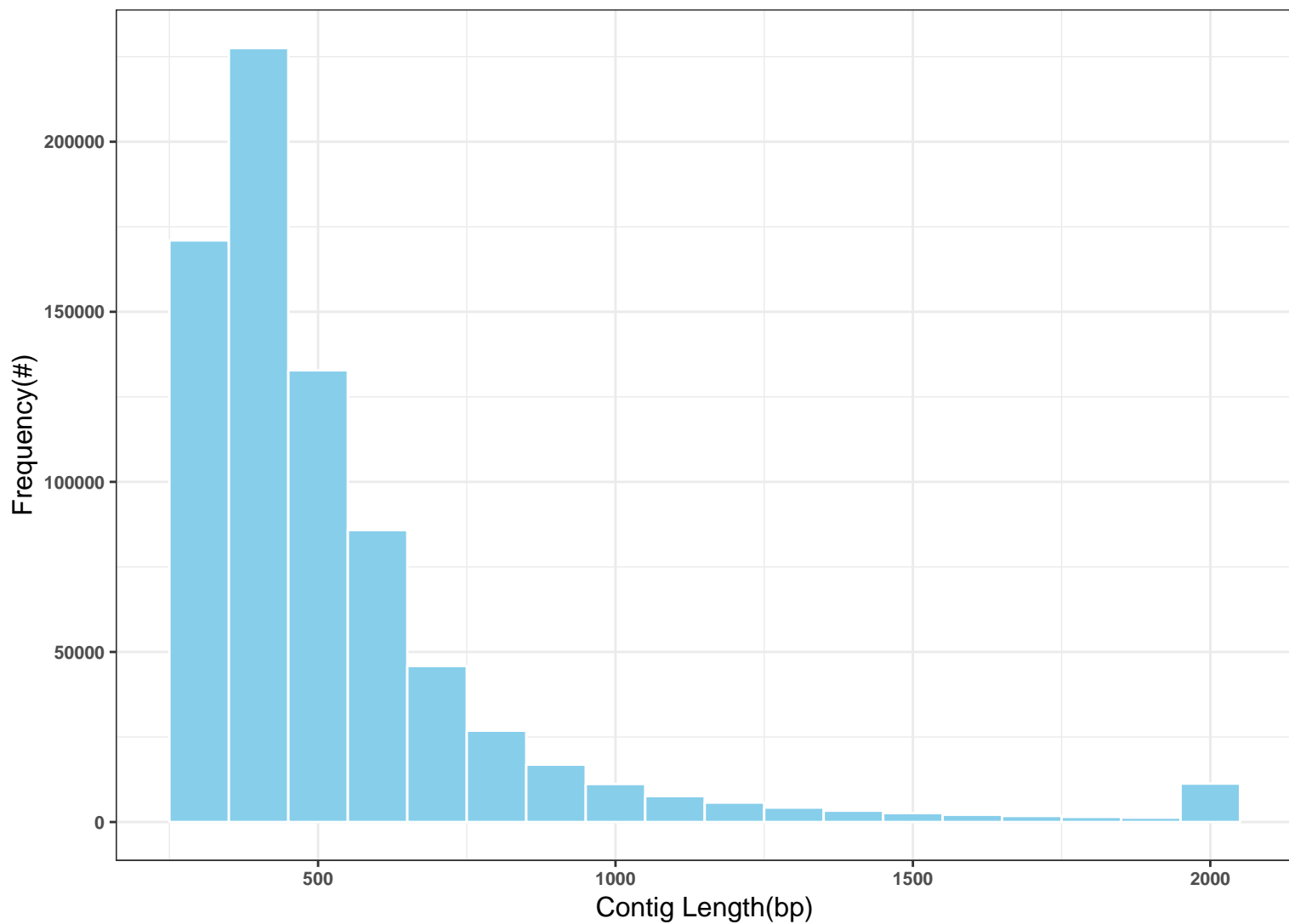

Supplement: Supplementary file 6 [file Image_1.pdf]

IFK3M1 Length Distribution

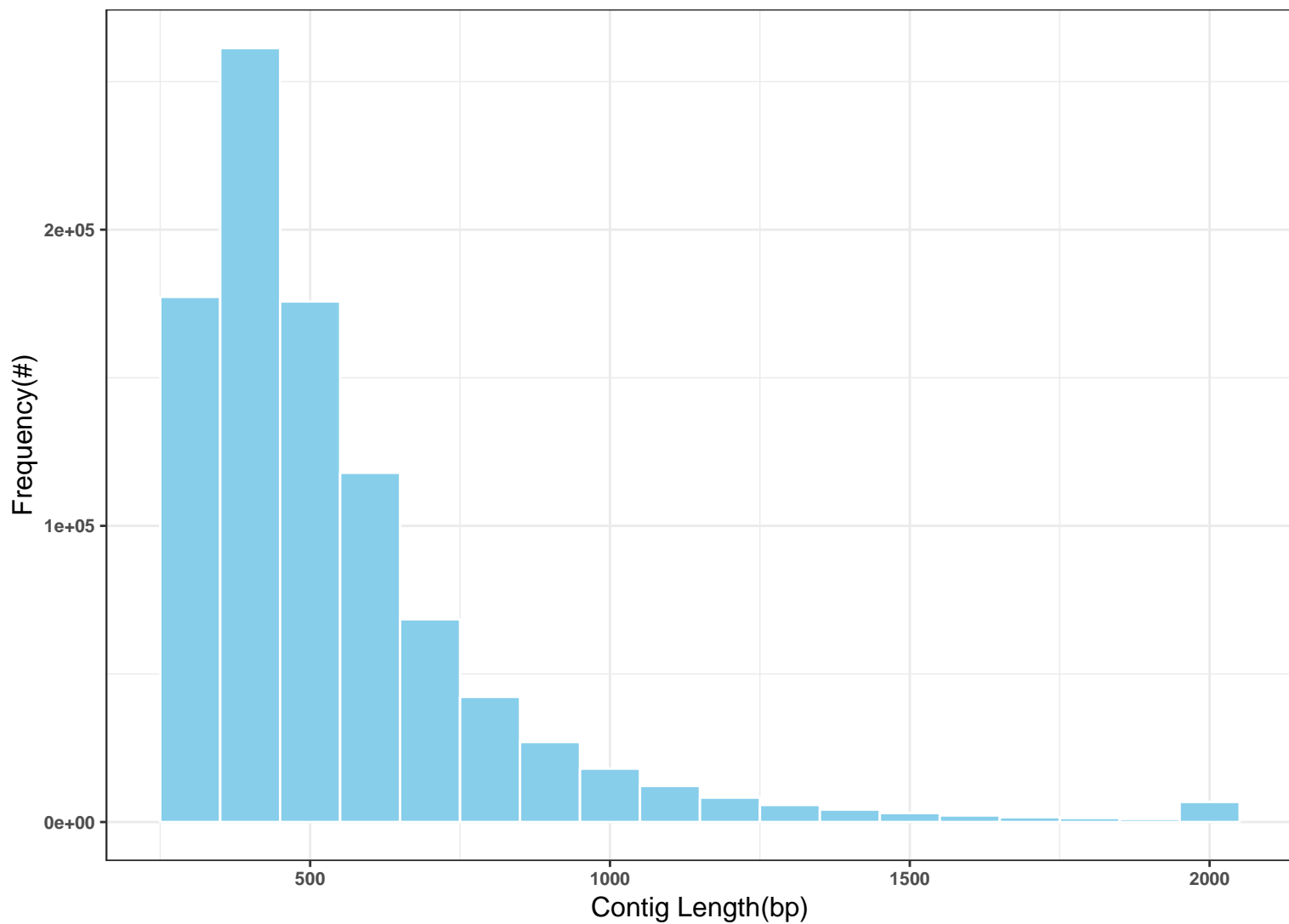

Supplement: Supplementary file 7 [file Image_2.pdf]

NF3M1 Length Distribution

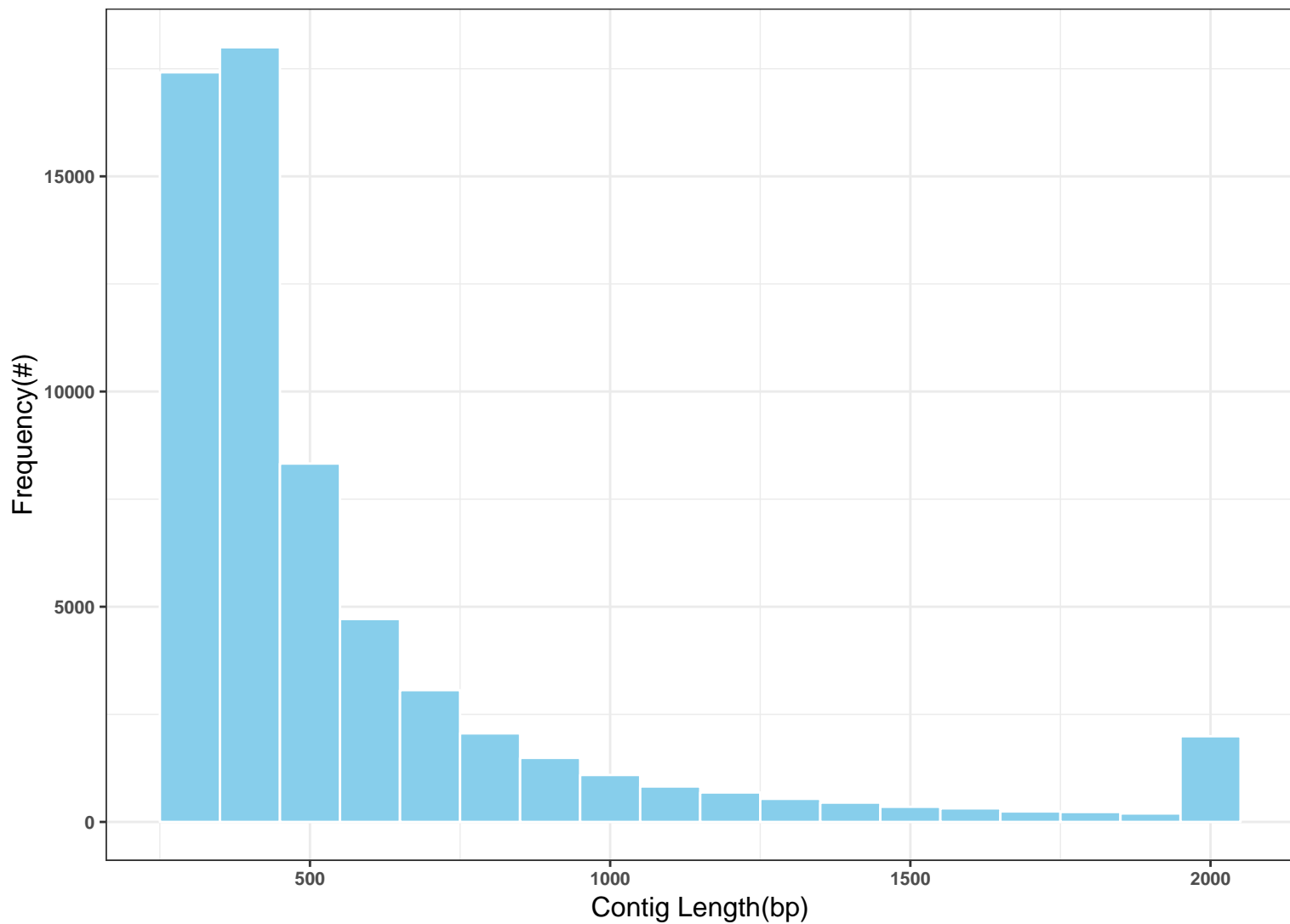

Supplement: Supplementary file 8 [file Image_3.pdf]

NFK3M1 Length Distribution

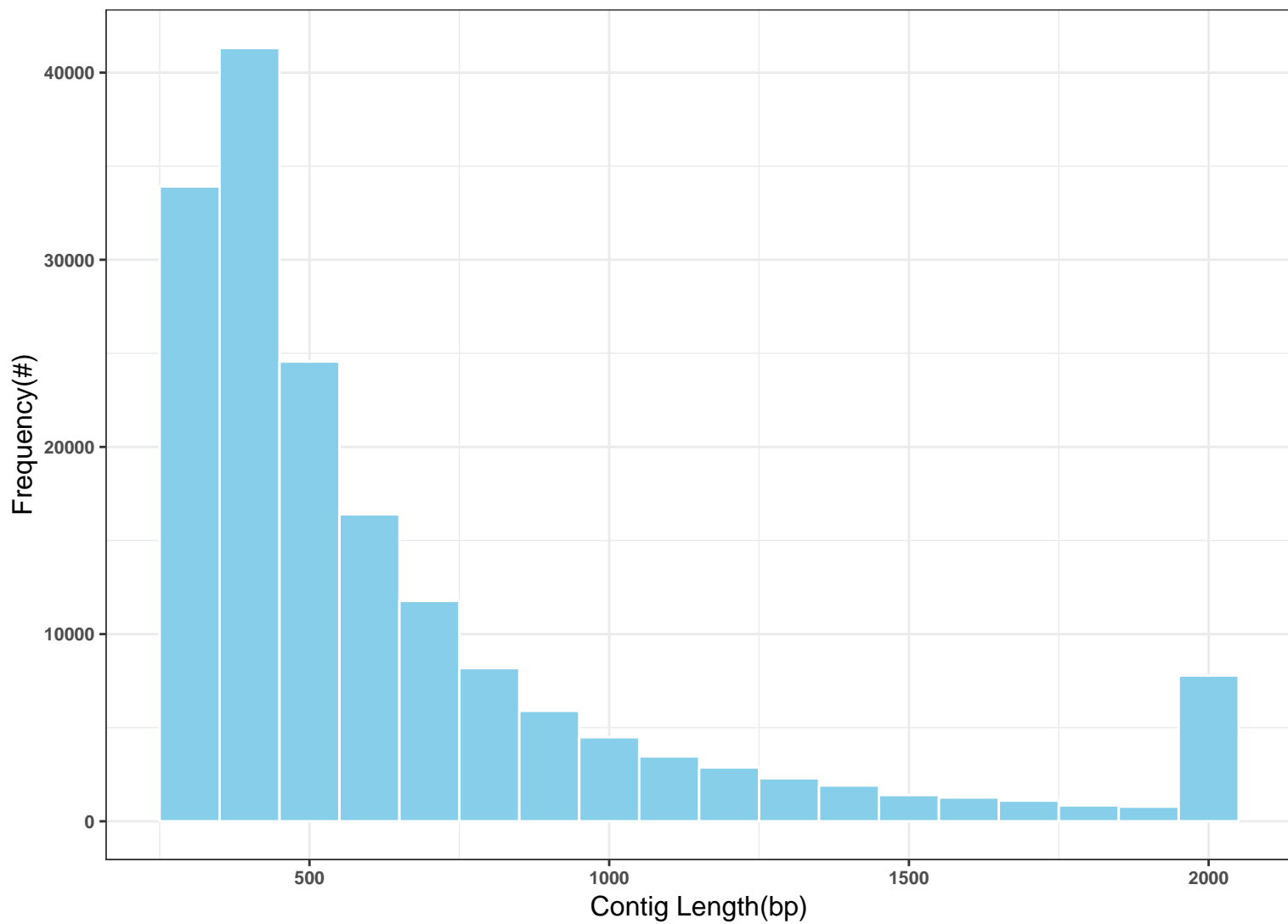

Supplement: Supplementary file 9 [file Image_4.pdf]
